# Neuronal Oscillations with Non-sinusoidal Morphology Produce Spurious Phase-to-Amplitude Coupling and Directionality

**DOI:** 10.3389/fncom.2016.00087

**Published:** 2016-08-22

**Authors:** Diego Lozano-Soldevilla, Niels ter Huurne, Robert Oostenveld

**Affiliations:** ^1^Cognition and Behaviour, Centre for Cognitive Neuroimaging, Donders Institute for Brain, Radboud University NijmegenNijmegen, Netherlands; ^2^Department of Psychiatry, Donders Institute for Brain, Cognition and Behaviour, Radboud University Medical CentreNijmegen, Netherlands; ^3^NatMEG, Department of Clinical Neuroscience, Karolinska InstitutetStockholm, Sweden

**Keywords:** cross-frequency coupling, neuronal oscillations, alpha rhythm, amplitude asymmetry, spectral analysis

## Abstract

Neuronal oscillations support cognitive processing. Modern views suggest that neuronal oscillations do not only reflect coordinated activity in spatially distributed networks, but also that there is interaction between the oscillations at different frequencies. For example, invasive recordings in animals and humans have found that the amplitude of fast oscillations (>40 Hz) occur non-uniformly within the phase of slower oscillations, forming the so-called cross-frequency coupling (CFC). However, the CFC patterns might be influenced by features in the signal that do not relate to underlying physiological interactions. For example, CFC estimates may be sensitive to spectral correlations due to non-sinusoidal properties of the alpha band wave morphology. To investigate this issue, we performed CFC analysis using experimental and synthetic data. The former consisted in a double-blind magnetoencephalography pharmacological study in which participants received either placebo, 0.5 or 1.5 mg of lorazepam (LZP; GABAergic enhancer) in different experimental sessions. By recording oscillatory brain activity with during rest and working memory (WM), we were able to demonstrate that posterior alpha (8–12 Hz) phase was coupled to beta-low gamma band (20–45 Hz) amplitude envelope during all sessions. Importantly, bicoherence values around the harmonics of the alpha frequency were similar both in magnitude and topographic distribution to the cross-frequency coherence (CFCoh) values observed in the alpha-phase to beta-low gamma coupling. In addition, despite the large CFCoh we found no significant cross-frequency directionality (CFD). Critically, simulations demonstrated that a sizable part of our empirical CFCoh between alpha and beta-low gamma coupling and the lack of CFD could be explained by two-three harmonics aligned in zero phase-lag produced by the physiologically characteristic alpha asymmetry in the amplitude of the peaks relative to the troughs. Furthermore, we showed that periodic signals whose waveform deviate from pure sine waves produce non-zero CFCoh with predictable CFD. Our results reveal the important role of the non-sinusoidal wave morphology on state of the art CFC metrics and we recommend caution with strong physiological interpretations of CFC and suggest basic data quality checks to enhance the mechanistic understanding of CFC.

## Introduction

Traditionally brain rhythms have been divided within different frequency bands: delta (1–4 Hz), theta (4–8 Hz), alpha (8–12 Hz), beta (15–30 Hz), and gamma (>30 Hz). These bands have been linked to a variety of normal and pathological behavioral phenotypes (Steriade et al., 1990). Importantly, this classification seems to be well-preserved amongst species despite the large differences in brain size (Buzsáki et al., 2013). Recent studies suggest that different brain rhythms can interact forming the so called cross-frequency coupling (CFC) (Jensen and Colgin, 2007). For example, a seminal work found that broadband gamma power (80–200 Hz) locked to theta rhythm in humans under various tasks (Canolty et al., 2006). In rat hippocampus beta-low gamma power (20–40 Hz) seems to be enhanced is specific phase bins of ongoing theta (Igarashi et al., 2014). Invasive recordings in animals and humans have implicated CFC dynamics to a large variety of functions such as neuronal communication between brain regions (Colgin et al., 2009; von Nicolai et al., 2014) and cortical layers (Spaak et al., 2012), regulation of the firing rate of a particular brain area (Canolty et al., 2012), associative learning (Tort et al., 2009; Igarashi et al., 2014), working memory (WM) information maintenance (Siegel et al., 2009; Axmacher et al., 2010) and neuronal communication of dispersed functional cell assemblies (Canolty et al., 2010, 2012). The overarching idea behind CFC is that independent brain rhythms interact hierarchically: while low frequency oscillations would synchronize large neuronal ensembles, local populations could be synchronized by faster rhythms modulated by the phase of slower oscillations (Buzsaki, 2006). Therefore, it is important to characterize the CFC to understand the dynamics and functions of brain rhythms.

Despite the popularity of the CFC framework, recent studies demonstrated potential caveats and major pitfalls in the interpretation of the results. Kramer et al. (2008) demonstrated that sharp transients inserted in periodic signals produce spurious (i.e., non-zero CFC), which resembles broadband gamma activity. The broadband activity arises because sharp-edged transients in the time domain correspond to high-frequency broadband harmonic components in the frequency domain. This phenomenon has subsequently been reported in rat hippocampus (Tort et al., 2013) (see Figure 8C of the paper). The term spurious CFC refers to the correlations between multiple frequency components (i.e., terms of the Fourier decomposition) caused when the input signal contains non-sinusoidal properties. These non-sinusoidal features can be transient or periodic and result in spectral correlations that will produce a non-zero CFC, even when a true physiological interaction is absent. More recently, Aru et al. (2015) provide an exhaustive review about potential caveats and confounds in the current CFC methodology, such as non-stationarities that may lead to spurious CFCs due to spectral correlations. They describe the difficulty to generate surrogate data that effectively destroys the specific cyclo-stationarities associated to the CFC effect of interest, while retaining the original probability distributions of all unspecific effects (i.e., not associated to the hypothesized CFC). Although the authors do not provide conclusive solutions to deal with the caveats, they propose a number of sanity checks to be performed in order to improve the mechanistic interpretation of the CFC.

Considering the caveats reviewed above (Kramer et al., 2008; Tort et al., 2013; Aru et al., 2015), the phase-to-amplitude coupling may overlap with the spectral harmonics of the alpha frequency. Various studies have shown (Stam et al., 1999; Nikulin et al., 2007, 2010) or observed (Gastaut, 1952; Contreras et al., 1997) that the alpha rhythm does not appear to be a pure sinusoidal wave, with a stronger amplitude of the peaks relative to the troughs. These non-sinusoidal features present in alpha oscillations will be expressed as harmonic components. On that note, harmonic components are, by definition, phase-to-amplitude coupled: the phase of the fundamental is locked to the power of the harmonic(s). This raises the concern whether the CFC patterns recorded in a given sensor are a genuine interaction reflecting a mechanistic physiological process between two independent rhythms, or whether it might be a more trivial consequence of spectral correlations due to the non-sinusoidal morphology of the alpha oscillations.

To investigate the influence of the morphology of neuronal oscillation on CFC metrics, we analyze human magnetoencephalographic (MEG) recordings during resting state and WM. We performed a double-blind randomized cross-over design pharmacological challenge where healthy participants received either placebo or lorazepam (LZP; GABA_A_ allosteric modulator) of different dosages (0.5 and 1.5 mg) in separate sessions. Pharmacological power modulations during WM have been published elsewhere (Lozano-Soldevilla et al., 2014). In addition, we constructed synthetic data varying systematically the shape of the signal to study its consequences with CFC metrics such as cross-frequency coherence (CFCoh), cross-frequency directionality (CFD), and bicoherence.

## Materials and methods

### Experimental procedures

Participants (*n* = 25) gave informed consent approved by the local research ethics committee (Commissie Mensgebonden Onderzoek; Arnhem-Nijmegen, number: 2011/199, date: August 5, 2011) and were compensated for their participation. They were engaged in a classic delayed-match to sample visuo-spatial WM task (Vogel and Machizawa, 2004) (Figure S1A) while brain oscillatory activity was acquired with whole-head MEG. Each trial started by presenting a centrally presented visual cue for 1.5 s. The task required participants to encode a sample array composed of colored squares presented in the visual hemifield indicated by a spatial cue. After a 1.5 s delay interval, a probe array was presented and participants had to indicate whether it matched the sample array or not. Participants had to keep their fixation to the center of the screen while covertly attend to the cued hemifield during the whole trial. Before the WM task, 2 min eyes-closed (EC) and 2 min eyes-opened (EO) resting state recordings were acquired (the order was counterbalanced across sessions).

### MEG acquisition and data analysis

Specific information about MEG data acquisition can be found in Lozano-Soldevilla et al. (2014) and we describe here the fundamental information. Ongoing brain activity was recorded using a whole-head MEG system with 275 axial sampled at 1200 Hz. To minimize participants head movements while maximizing their comfort, we used an orthopedic neck-collar during the MEG recordings (Push braces, height 10, size 2, model 1.60.2.02; http://www.push.eu). We used the real-time head localizer tool (Stolk et al., 2013) to monitor participant's head position and orientation relative to the MEG sensors during all the recordings.

MEG data was low-pass filtered below 200 Hz and down-sampled to 600 Hz. Subsequently we analyzed our dataset using Fieldtrip (Oostenveld et al., 2011) (http://fieldtriptoolbox.org); an open-source toolbox developed at the Donders Institute for Brain, Cognition, and Behaviour (Nijmegen, The Netherlands) and custom Matlab code (MathWorks, Natick, MA, USA).

#### Sensor analysis

##### Resting-state data

We eliminated line noise by fitting sine and cosine functions between 48–52, 98–102, 148–152 Hz and subsequently subtracting these estimated components. EC and EO recordings were cut in epochs of 120 s duration. Raw data was visually inspected and the pieces containing eye-blink, muscle activity, or sensor artifacts were rejected. Sensors with noisy periods were rejected and linearly interpolated (neighbor average). After partial artifact rejection, data segments shorter or equal to 2 s were excluded leaving the rest of the cleaned data for the main analysis. Remaining eye movements and electrocardiogram (ECG) activity were isolated by independent component analysis (ICA). The independent components (ICs) reflecting potential artifacts were semi-automatically identified by correlating its time-course with the respective vertical EOG and ECG bipolar derivations. Given the stable periodicity of the QRS complex sharp edges, spurious CFC could arise (Kramer et al., 2008). To reduce ECG residual activity after the removal of the identified ICs, we adapted a procedure proposed by Tal and Abeles (2013). Briefly, we band-pass filtered the ECG with a zero-phase two-pass 4th order Butterworth filter (0.5–50 Hz). Then we took the absolute value of the ECG signal and we found the QRS peaks separated by a minimum of 0.66 s. Once all the peaks were identified, we computed peak-triggered averages (–0.3 to 0.4 s) using the time points defined in the ECG bipolar channel of the unfiltered MEG data. The obtained QRS-template was subtracted from the MEG recordings cycle-by-cycle. We observed QRS residuals in most of the participants after ICA nonetheless. Given that it has been empirically demonstrated that the reliability of the Infomax algorithm correlated positively to the amount of data samples used during the decomposition and it correlated negatively to the number of sensors^2^ (Groppe et al., 2009), it is likely that the Infomax did not have enough data points per sensor to perform a more accurate solution (i.e., we only obtained < 4 min recordings with 273 sensors).

Spectral oscillatory power was performed by means of Fast Fourier Transform (FFT) using a fixed window length of 4 s (4–40 Hz in 0.25 Hz steps), 50% overlap, multiplied by a Hanning taper. Prior to spectral analysis, the mean of each epoch was subtracted. Spectra were normalized based on the total power in each sensor within each participant and MEG session before group averaging.

To investigate cross-frequency interactions we chose the CFCoh index (Osipova et al., 2008). CFCoh measures the phase consistency between a low frequency signal and the amplitude envelope of higher frequencies. Let the signal be represented by the time series *x*_1_, *x*_2_, …, *x*_*N*_ where *N* is the number of time points acquired at specific sampling rate (*Fs*). First, the time-course of amplitude $y^{\nu }=\left(y_{1}^{\nu },\;y_{2}^{\nu },\ldots ,y_{N}^{\nu }\right)\;$ was estimated for frequency *v* by applying a sliding tapered time-window followed by a Fourier transform:

(1) $$\begin{array}{l} y_{n}^{v}=\frac{1}{Fs}|\sum_{m=1}^{M}h_{m}x_{n+m-M/2}e^{\frac{-i2\pi vm}{Fs}}| \end{array}$$

The function *h*_*m*_ is a Hanning taper *M* data points-long equaling the length of the sliding time window. The length of the time-window decreases with frequency such that *M* = *w* · *Fs*/*v*, where *w* denotes the numbers of cycles per time window (five cycles in the present study). Note that the time-series ***x*** can be zero-padded to reduce boundary effects at the beginning and end of the segments. In the present analysis, low frequencies were defined between 5 and 25 Hz and higher frequencies from 5 to 90 Hz in steps of 1 Hz.

Next we proceeded to estimate the Fourier spectra of ***x*** and ***y***. Data was segmented into a set of *S* segments 0.6 s long with 50% overlap. Using a standard FFT algorithm, the complex representations were calculated:

(2) $$\begin{array}{l} X^{s}=\text{FFT}\left(h^{T}x^{s},n_{FFT}\right) \end{array}$$

and

(3) $$\begin{array}{l} Y^{\upsilon ,s}=\text{ FFT}\left(h^{T}y^{v,s},n_{FFT}\right) \end{array}$$

with ***x***^*s*^ representing the temporal evolution of raw signal of segments *s*, ***y***^*v, s*^ representing the amplitude envelope at high frequency *v* and segment *s* and *n*_*FFT*_ representing the frequency resolution of the x-axis Δ*f* = *Fs*/*n*_*FFT*_ of the CFCoh comodulogram (*n*_*FFT*_ = 1024). The output vector $X^{s}=\left(X_{1}^{s},X_{2}^{s},\ldots ,X_{n_{FFT}}^{s}\right)$ and $Y^{v,s}=\left(Y_{1}^{v,s},Y_{2}^{v,s},\ldots ,Y_{n_{FFT}}^{v,s}\right)$ were Fourier transforms centered at frequencies: $f\in \left\{0,\frac{Fs}{n_{FFT}},\ldots ,\frac{Fs}{2}\left(1-\frac{1}{n_{FFT}}\right)\right\}$. The cross-spectra **ξ** of the individual segments was defined as **ξ**^*v, s*^ = ***X***^*s*^(***Y***^ν, *s*^)^*^ where “*” denoted the complex conjugate.

To quantify the CFC, we used the magnitude squared CFCoh (**η^*2*^**):

(4) $$\begin{array}{l} \eta^{2}(v,f)=\frac{{\left|\sum_{s\;=\;1}^{S}\xi^{v,s}\right|}^{2}}{\sum_{s\;=\;1}^{S}{\left|X^{s}\right|}^{2}\cdot \sum_{s\;=\;1}^{S}{\left|Y^{v,s}\right|}^{2}} \end{array}$$

where frequencies were defined as $f\in \left\{0,\frac{Fs}{n_{FFT}},\ldots ,\frac{Fs}{2}\left(1-\frac{1}{n_{FFT}}\right)\right\}$.

To assess the directionality of CFCoh, we employed the new CFD metric (Jiang et al., 2015). CFD is the CFC extension of the phase-slope index (PSI) (Nolte et al., 2008). It rests on the assumption that when the transmission between two signals (sender and receiver) is associated with a fixed delay, the phase difference between the signals will change systematically with frequency as observed in the phase spectrum (Gotman, 1983; Mima et al., 2000). The PSI estimates the slope of the phase difference as a function of frequency in a given band with the direction of interaction attached to its sign. We here applied the PSI to the signal ***x*** and the amplitude envelope of the signal ***y***^*v*^ at frequency tile (*v, f*_*j*_):

(5) $$\begin{array}{l} \psi \;\left(v,f_{j}\right)=ℑ\;\left(\sum_{f_{j}\;-\;\frac{\beta }{2}}^{f_{j}\;+\;\frac{\beta }{2}}C^{*}\left(v,f_{j}\right)\;C\;\left(v,\left(f_{j}+\Delta f\right)\right)\right) \end{array}$$

where the complex coherency was defined as:

(6) $$\begin{array}{l} C\left(v,f_{j}\right)=\frac{\sum_{s=1}^{S}X^{s}{\left(Y^{v,s}\right)}^{*}}{\sqrt{\sum_{s=1}^{S}{\left|X^{s}\right|}^{2}\sum_{s=1}^{S}{\left|Y^{v,s}\right|}^{2}}} \end{array}$$

with Δ*f* denoting the frequency resolution and ℑ() representing the imaginary part. We used β to denote the bandwidth for which the phase slope was calculated (β = 4 Hz). $\tilde{\Psi }$ corresponded to the weighted average of the slope and we normalized it by an estimate of its standard deviation through a jackknife procedure (i.e., Ψ = $\tilde{\Psi }$*/std(*$\tilde{\Psi }$*)*). The authors considered absolute values larger than 2 as significant (Nolte et al., 2008).

In order to assess cross-frequency phase-to-phase coupling we computed the magnitude squared bicoherence index (Kim and Powers, 1979):

(7) $$\begin{array}{l} b^{2}\left(v,f\right)=\;\frac{{\left|\sum_{s=1}^{S}X^{s}\left(v\right)X^{s}\left(f\right)X^{*s}\left(v+f\right)\right|}^{2}}{\sum_{s=1}^{S}{\left|X^{s}\left(v\right)X^{s}\left(f\right)\right|}^{2}\;\sum_{s=1}^{S}{\left|X^{s}\left(v+f\right)\right|}^{2}} \end{array}$$

The bicoherence is close to 0 when the coherence of Fourier frequency triplets (*v*, *f*, and *v* + *f*) behave randomly across *S* segments and it is close to 1 when the phase of the numerator (bispectrum) remains constant over segments. As for CFCoh, we epoched the data using fixed window length of 0.6 s, 50% overlapping, multiplied by a Hanning taper.

To further explore the phasic relationship between beta-low gamma power and the alpha rhythm, we took two complementary approaches based on event-related averages. The first one was based on the computation of time-frequency representations (TFR) locked to the alpha peak (Canolty et al., 2006; Osipova et al., 2008). To archive that, the MEG axial gradiometers of each participant and pharmacological session were band-pass filtered within the alpha band (8–12 Hz; zero-phase two-pass 4th order Butterworth filter). Alpha peaks were identified and the unfiltered raw data was cut in 0.6 s epochs centered at the peak. TFRs of power were calculated for each single trial using same parameters as for the CFCoh (i.e., ${\left|y^{v}\right|}^{2}$). Induced power estimates were averaged across trials and normalized relative to the mean power per frequency. Complementarily, we also performed event-related averages taking the gamma peak-as a trigger (Bragin et al., 1995).

##### Resting state source analysis

To localize the LZP-related oscillatory power modulations during EC and EO epochs, we adopted a beamformer approach as defined in Gross et al. (2001). Alpha band source reconstruction was computed by applying a FFT approach (epochs of 0.6 s) centered at 10 Hz using a one Slepian taper resulting in 2 Hz smoothing. The source reconstruction of the beta band activity (14–30 Hz) was centered at 22 Hz and 9 orthogonal Slepian tapers resulting 8 Hz smoothing. The brain volume of each individual was divided into a grid with a 1 cm resolution and. For every grid point a spatial filter was constructed from the lead field and the cross-spectral density (CSD) matrix. The lead fields were calculated from a subject specific realistic single-shell model of the brain (Nolte, 2003). CSD matrices were computed between all MEG pairs for the time period and frequency of interest. Experimental conditions (EC, EO) were combined for the purpose of calculating a common spatial filter, after which the power at each grid point was estimated for each session separately in every participant. We estimated the orientation in which the power of each dipole grid was maximal according by single value decomposition (Schoffelen et al., 2011). Prior to averaging, the source estimates of the individual functional data were normalized onto the MNI brain template (International Consortium for Brain Mapping, Montreal Neurological Institute, Canada) using SPM8 (http://www.fil.ion.ucl.ac.uk/spm).

##### Working memory (WM) data

Preprocessing, artifact rejection, and sensor spectral analysis procedures were done as in Lozano-Soldevilla et al. (2014). Briefly, trials of 1 s (0.5–1.5 s after memory sample array) were extracted for each pharmacological session. CFCoh, CFD and bicoherence sensor analysis were performed on MEG axial gradiometers with same parameters as those ones used for the resting state data.

##### Statistical analysis

To statistically assess the drug main effect on oscillatory power, CFCoh, bicoherence, or peak-triggered averages at the sensors space, a cluster-based nonparametric permutation test was applied (Maris and Oostenveld, 2007). This approach provides an effective method for controlling for multiple comparisons.

A repeated-measures ANOVA approach was used to asses drug main effects on selected sensors of interest. *P*-values were calculated and Greenhouse-Geisser corrections were made to control of sphericity violation when appropriated. Subsequent *post-hoc t*-tests, in case of main effect and/or interaction, were always corrected by multiple comparisons using the false discovery rate approach (Benjamini and Hochberg, 1995).

### Simulations

We constructed synthetic data to demonstrate the role of signal wave morphology on different CFC metrics. All simulations were generated using a sampling rate of 1000 Hz and 180 s duration.

#### Narrow gamma amplitude (60–80 Hz) to alpha phase (~10 Hz) CFC

We created a signal containing CFC between the alpha and gamma by adapting the simulation described in example 3 from Kramer et al. (2008):

(8) $$\begin{array}{l} \alpha =\cos\left(2\pi ft\right) \end{array}$$

where the α frequency *f* randomly varied cycle-by-cycle with the form *f* = 1/(0.1 s ± 0.01 s). In other words, we introduced small temporal variations in its period. The variable *t* defined the time axis running from 0 to 1 s. To each cycle of the oscillation we add a short 50 ms burst of high frequency (60–80 Hz) white noise that was tapered with a Hanning window. The Hanning window was scaled to a maximum amplitude of 5. The onset of the gamma signal relative to the alpha cycle varied uniformly between 0 and 20°C. Finally, normally distributed random noise with zero mean and standard deviation 0.1 were added.

#### Narrow-band gamma amplitude (60–80 Hz) to alpha phase CFC with non-sinusoidal wave morphology properties

We generated the same alpha-gamma CFC as in (1) but this time the α waveform was generated with non-sinusoidal wave morphology properties. Concretely, the magnitude of the amplitude of the peaks of the alpha oscillations was higher relative to the troughs:

(9) $$\begin{array}{l} \alpha ={\left(\left(\cos\left(2\pi ft\right)+1\right)/2\right)}^{b} \end{array}$$

This resulted in signals with a “Gaussian-like” wave morphology, where the exponent *b* controlled the degree of amplitude asymmetry of peaks relative to the troughs. The exponent *b* was chosen to be 3. The signal α was demeaned and rescaled by a factor of 2. Note that the properties of the non-sinusoidal signal morphology described above differ from the amplitude fluctuation asymmetry properties defined by Mazaheri and Jensen (2008). Amplitude fluctuations presented in this manuscript do not refer to the normalized variance of the peaks and troughs. The alpha morphology model defined here based on the assumption that the magnitude of the amplitude of the peaks (troughs) was higher relative to the magnitude the troughs (peaks). Critically, the variance of the peaks/troughs in our model was highly similar (did not change over time) although the asymmetry in the magnitude of the peaks/troughs was very strong (see Discussion Section).

#### Beta-low gamma amplitude harmonics (20–40 Hz) coupled to alpha phase

We simulated a “sawtooth” signal of 10 Hz period with and without frequency fluctuations using the following Equation (Maccarone, 2013):

(10) $$\begin{array}{l} \alpha =\sum_{j\;=\;1}^{J}\frac{1}{j^{2}}\;\cdot \;cos\left(j2\pi ft+\left(j-1\right)\;\cdot \;\varphi \right) \end{array}$$

where *j* indexed the *j*-th harmonic of frequency *f*, and the phase term (*j* − 1) · φ determined the phase of the *j*-th harmonic. We defined the first two harmonics of the alpha band (20 and 30 Hz, *J* = 3). The sum of the fundamental and the 1st and 2nd harmonics with the phase term φ = π/2 generated a sawtooth wave whose amplitude raised rapidly decreasing with time in a given cycle (“left-side peak”). This waveform was referred as “negative sawtooth” signal. In contrast, we refered to a “positive sawtooth” (“right-side peak”) a waveform with a constant raising period and a rapidly amplitude decrease (φ = 3π/2). We explored different φ-values [2π, π/4, π/2, 3π/4, π, 5π/4, 3π/2, 7π/4] to investigate systematically a variety of periodic wave morphologies. In comparison to Kramer et al. (2008), who showed spurious CFC due to the sharp edges in a sawtooth signal, we used the $\frac{1}{j^{2}}$ factor, which results in a more curved sawtooth signal to avoid high broadband frequency activity in the power spectrum (Maccarone, 2013). Importantly, it has been not tested yet whether wave morphologies influences CFD (i.e., non-linear coupling by harmonics of non-sinusoidal oscillations) and under which circumstances this measure provides false positives (Jiang et al., 2015).

#### Effects of sharp edge artifacts on CFD

Adapted from example 2 in Kramer et al. (2008), we explored how sharp transitions introduced into each alpha cycle would affect not only the CFCoh and bicoherence measures, but critically CFD. We created a 10 Hz sinusoid with a constant period and we added sharp edges that linearly increased the amplitude of the oscillation after 1–5 ms (uniformly distributed over the cycles). The sharp edges began at phase values uniformly distributed between 233 and 265°C (cosine phase).

## Results

### LZP modulations in alpha (8–12 Hz) and beta (15–30 Hz) bands during resting state

Spectral analysis was applied to the MEG axial gradiometers to investigate the power modulations produced by LZP administration during the EO and EC conditions. We found significant differences between drug conditions both during EO (cluster-based nonparametric permutation test, *p* < 0.001; Figure S2A left) and during EC (*p* < 0.001; Figure S2A right). Under resting state conditions, LZP reduced alpha power in many sensors, especially in occipital areas. Repeated-measures ANOVA revealed that LZP strongly reduced occipital alpha power during EO [*F*_(2, 48)_ = 14.48, *p* < 5 × 10^−5^; Placebo = 0.5 mg LZP: *t*_(24)_ = 1.44; *p* = 0.16; Placebo > 1.5 mg LZP: *t*_(24)_ = 4.42, *p* < 0.001; 0.5 mg LZP > 1.5 mg LZP: *t*_(24)_ = 3.95, *p* < 0.001; Figure S2B top]. During EC there was also a robust posterior power reduction by LZP [*F*_(2, 48)_ = 7.66, *p* < 0.005; Placebo = 0.5 mg LZP: *t*_(24)_ = 1.24; *p* = 0.23; Placebo > 1.5 mg LZP: *t*_(24)_ = 4.24, *p* < 0.001; 0.5 mg LZP > 1.5 mg LZP: *t*_(24)_ = 2.45, *p* < 0.05; Figure S2B bottom]. To identify the brain sources associated the drug-related power modulations, we pooled the source data over the resting state conditions ((EO + EC)/2) and then considered the relative change: (1.5 mg LZP–Placebo)/Placebo. As expected, the maximum of the LZP alpha power decrease was located over the occipito-parietal cortex (Figure S2C), in line with the LZP-induced power modulations during WM (Lozano-Soldevilla et al., 2014).

While LZP decreased occipital alpha power, fronto-central beta power increased as a function of dosage (Figure S2D) both during EO [*F*_(2, 48)_ = 12.91, *p* < 5 × 10^−5^; Placebo = 0.5 mg LZP: *t*_(24)_ = −0.88; *p* = 0.38; Placebo > 1.5 mg LZP: *t*_(24)_ = −4.12, *p* < 0.001; 0.5 mg LZP > 1.5 mg LZP: *t*_(24)_ = −4.23, *p* < 0.001; Figure S2E top] and during EC [*F*_(2, 48)_ = 38.87, *p* < 1 × 10^−10^; Placebo > 0.5 mg LZP: *t*_(24)_ = −3.98; *p* < 0.001; Placebo > 1.5 mg LZP: *t*_(24)_ = −7.06, *p* < 1 × 10^−6^; 0.5 mg LZP > 1.5 mg LZP: *t*_(24)_ = −5.81, *p* < 1 × 10^−5^; Figure S2E bottom]. In line with previous evidence (Jensen et al., 2005; Hall et al., 2010), the LZP-induced beta power-increased was found over sensorimotor cortices (Figure S2F).

In conclusion, LZP produced robust power modulations during resting state conditions: while parieto-occipital alpha power decreased with LZP dosage, sensorimotor beta power increased.

### LZP decreased alpha-phase to beta-low gamma amplitude coupling during rest

**Figure 1:**
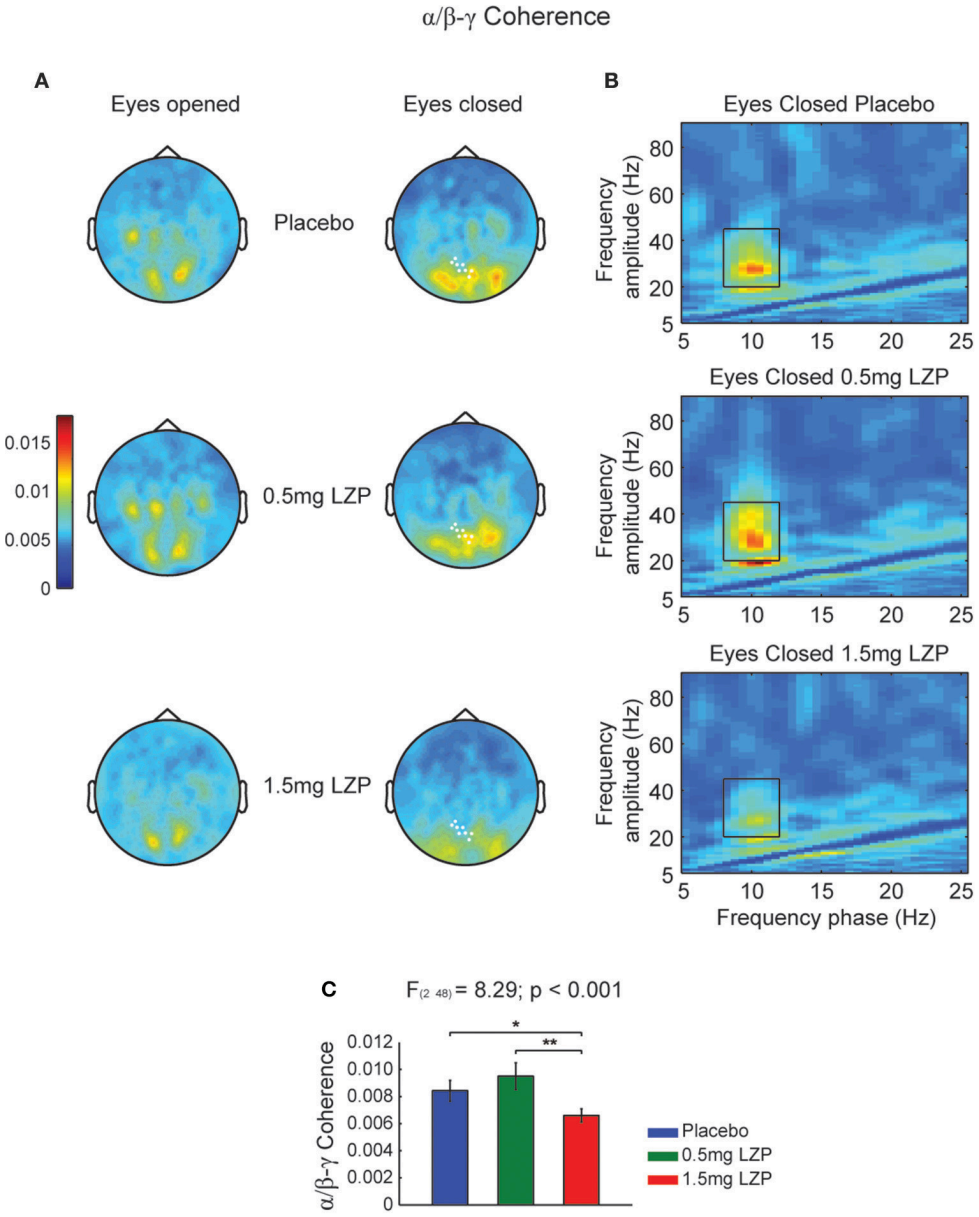
**High LZP dosage decreases alpha phase (8–12 Hz) to beta-low gamma (20–45 Hz) amplitude coupling during EC. (A)** Grand mean topographic representation of alpha phase to beta-low gamma amplitude coupling during EO (left) and EC (right) resting state. The color reflect the magnitude of coupling (magnitude squared coherence). The rows reflect the drug levels: Placebo, 0.5 and 1.5 mg LZP. The sensors marked in white displayed significant drug main effect (repeated-measures ANOVA; multiple comparisons controlled by a cluster-based nonparametric permutation test). **(B)** Grand mean frequency-by-frequency coherence comodulogram of the occipital axial gradiometers that were significantly modulated by LZP [white marked sensors in **(A)**]. Same color code and scale as in **(A)**. Black rectangles indicate frequency ranges used for the statistical analysis and topographic representations (8–12 Hz; 20–45 Hz). **(C)** 1.5 mg LZP decreased alpha phase to beta-low gamma amplitude coupling as revealed by repeated-measures ANOVA. ^**^p < 0.005; ^*^p < 0.05. Error bars show the SEM.

Given we found a clear alpha power peak in the resting state spectrum, we set out to investigate whether the ongoing alpha phase linked with higher frequency amplitude modulations. Moreover, we also sought whether LZP modulated its amplitude or frequency. First, we calculated the CFCoh metric for all participants and drug sessions during rest. We observed alpha phase (8–12 Hz) to beta-low gamma (20–45 Hz) amplitude coupling over posterior sensors (Figure 1A). Moreover, LZP significantly changed the alpha/beta-low gamma CFC during EC conditions (cluster-based nonparametric permutation test, *p* < 0.001; Figure 1A right, marked sensors) but not during EO. Repeated-measures ANOVA on sensors of interest revealed that GABAergic enhancement decreased alpha/beta-low gamma CFC under high LZP dosage relative to Placebo and low dosage, with no significant differences between Placebo and low dosage [*F*_(2, 48)_ = 8.29, *p* < 0.001; Placebo = 0.5 mg LZP: *t*_(24)_ = 1.42; *p* = 0.17; Placebo > 1.5 mg LZP: *t*_(24)_ = 2.81, *p* < 0.05; 0.5 mg LZP > 1.5 mg LZP: *t*_(24)_ = 3.85, *p* < 0.005; Figures 1B,C]. It is interesting to observe that the beta-low gamma amplitude (Figure 1B) was not detected in the classical spectral analysis (Figure S2B).

**Figure 2:**
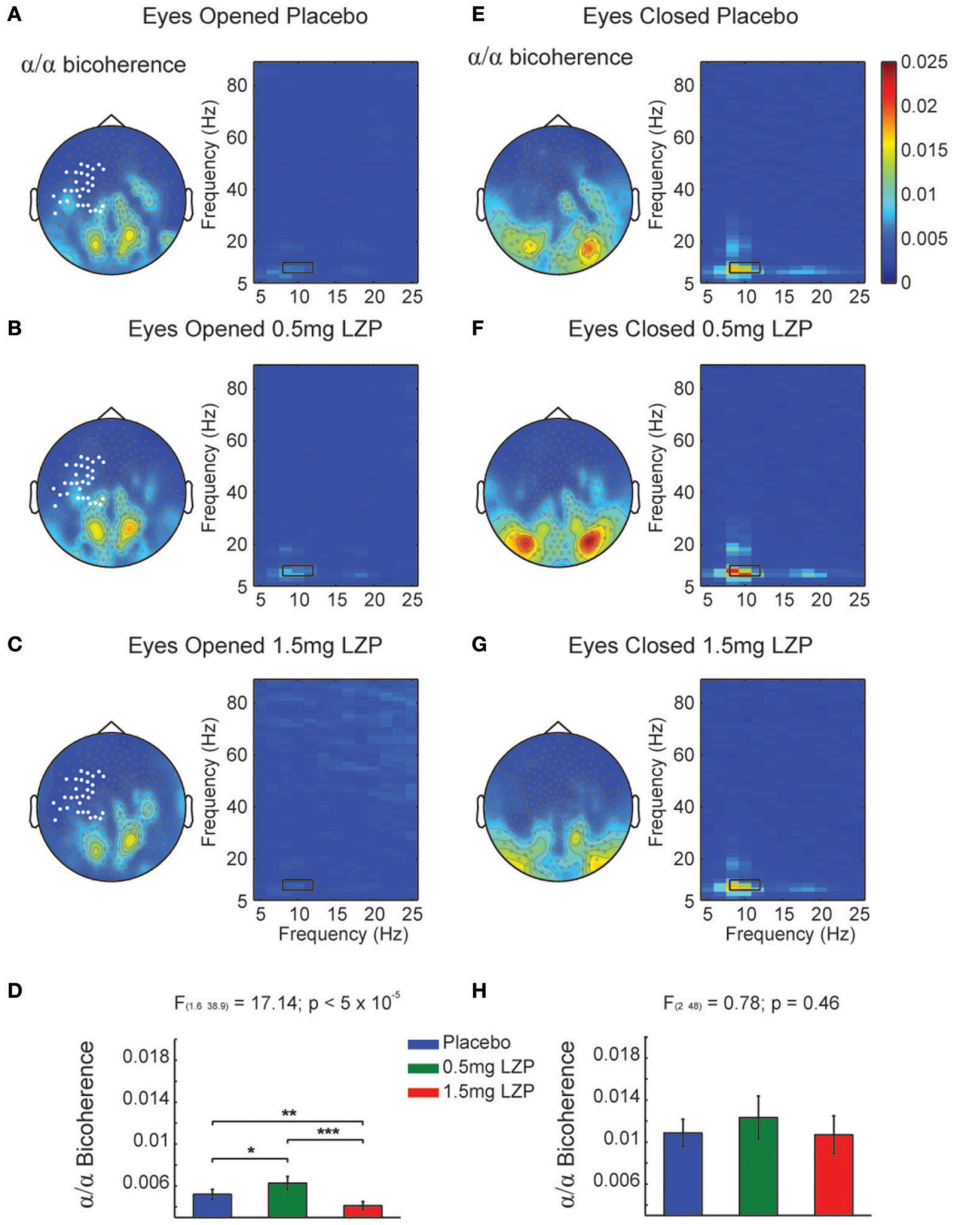
**Bicoherence modulations by LZP during EO and EC**. **(A)** Grand mean topographic representation of alpha (8–12 Hz) to alpha coupling under Placebo during EO (left) and comodulogram (right). Color code represents magnitude squared bicoherence. Black rectangles in the comodulogram indicate frequency ranges used for the statistical analysis and topographic representations (8–12 Hz; 8–12 Hz). White marked sensors over the left sensorimotor cortex indicate a statistically significant drug main effect (cluster-based nonparametric permutation test, *p* < 0.005). **(B)** Topographic representation of bicoherence (left) and comodulogram (right) under 0.5 mg LZP during EO [same conventions as **(A)**]. **(C)** Topography representation of bicoherence (left) and comodulogram (right) under 1.5 mg LZP during EO [same conventions as **(A)**]. **(D)** LZP modulated sensorimotor (white bold marked sensors) alpha bicoherence in an inverted U-shape as revealed by repeated-measures ANOVA. Error bars show the SEM. **(E)** Grand mean topographic representation of alpha to alpha coupling under Placebo during EC (left) and comodulogram [right; same conventions as **(A)**]. The comodulogram displays the average over the occipital white marked sensors from **(A)**]. **(F)** Topography and bicoherence comodulogram under 0.5 mg LZP during EC [same conventions as **(B)**]. **(G)** Topography and bicoherence comodulogram for 1.5 mg LZP during EC [same conventions as **(C)**]. **(H)** LZP did not modulated occipital alpha bicoherence coupling [white bold marked sensors topography in **(A)**]. ^***^p < 5 × 10^−4^; ^**^p < 0.005; ^*^p < 0.01. Error bars show the SEM.

It could be that the CFCoh we observe in Figure 1 were produced by harmonics of the alpha oscillations. To test this possibility we computed the magnitude squared bicoherence index for the MEG axial gradiometers. We observed strong bicoherence values within the alpha range during EO peaking over left central and parieto-occipital sensors. The former (left central sensors) were modulated by LZP (cluster-based nonparametric permutation test, *p* < 0.005; Figures 2A–C left, marked sensors) showing an inverted U-shape [*F*_(1.6, 38.9)_ = 17.14, *p* < 5 × 10^−5^; Placebo < 0.5 mg LZP: *t*_(24)_ = −3.02; *p* < 0.01; Placebo > 1.5 mg LZP: *t*_(24)_ = 3.73, *p* < 0.005; 0.5 mg LZP > 1.5 mg LZP: *t*_(24)_ = 4.88, *p* < 5 × 10^−4^; Figure 2D]. Posterior sensors did not show statistical significant differences as a function of drug dosage. During EC conditions, non-significant differences were found (cluster-based nonparametric permutation test, *p* = 0.22; Figures 2E–H) but strong bicoherence values were observed over posterior axial gradiometers around ~10 and ~20 Hz. On that note, the CFCoh topographies (Figure 1A) in both resting state conditions remarkably overlapped to both bicoherence (Figure 2) and alpha power topographies (Figure S1A). It is worth to mention that the bicoherence values in the alpha range (8–12 Hz x-axes; 8–12 Hz y-axes; Figure 2, black rectangles) indicated that, over occipital sensors, there were not only consistent phase differences within the ~10 Hz frequency but also between the ~10 and the ~20 Hz frequencies (i.e., ${\left|\overset{S}{\underset{s=1}{\sum }}X^{s}\left(v\right)X^{s}\left(f\right)X^{*s}\left(v+f\right)\right|}^{2}$), suggesting three harmonics in the power spectrum.

In summary, parieto-occipital sensors display the strongest phase-to-phase coupling between alpha and its first two harmonics (Figures 2E–G right side).

Figure 1 indicated that there was a CFCoh between alpha and beta-low gamma. Next we asked whether the phase of alpha oscillations drove the power of beta-low gamma oscillations or conversely whether the power of beta-low gamma drove the phase of the alpha oscillations. In addition, a third possibility could appear on where the phase of alpha and the power of beta-low power would interact around zero phase-lag. To achieve that, we used the new CFD metric on MEG axial gradiometers for all participants and sessions during EC. Figure S3A showed the topographic representation of the CFD index (Jiang et al., 2015) over the same frequency ranges as in Figure 1A. We did not observe a clear directionality pattern over posterior sensors. Also, we did not observe significant drug modulations taken our frequency regions of interest (8–12 Hz; 20–45 Hz; cluster-based nonparametric permutation test, *p* = 1). The CFCoh observed in Figure 1B was not present in the CFD comodulogram in any of the MEG sessions, with very low sigma values ± 0.3 (Figures S3B–D). CFD index did not show the CFCoh coupling pattern found in Figure 2.

The presence of harmonics in the data (Figure 2) could produce spurious CFCoh, raising the question whether the beta-low gamma power band was an independent oscillator or whether it came from the non-sinusoidal properties of the alpha band. To test between these possibilities, complementary CFC metrics were used. To assess the robustness of the beta-low gamma amplitude envelope to alpha phase coupling, we calculated the high frequency TFRs locked to the alpha peaks. If beta-low gamma power is phase locked to the alpha rhythm as the CFCoh index suggested, specific alpha phase segments (i.e., peaks or thoughts) should be associated with power modulations. Furthermore, those modulations should be differently affected by LZP as shown in Figure S2. Contrary to our hypothesis, we did not observe the beta-low gamma power modulations at any alpha cycle and it was not modulated by the drug levels (Figure S4). Despite the clear alpha waves whose central peak (time = 0s) were decreased with LZP (Figures S4D,E,I,J), there were not clear oscillatory beta-low gamma power modulations locked to neither alpha peaks nor troughs. Complementarily, we employed the event-related peak triggered averages around the beta-low gamma frequency range to align signal traces nested to slow oscillations. It is important to note that in case of robust CFC, the high frequency amplitude peak traces (time = 0 s) should be on the top of the specific phase of the ongoing oscillation. Despite the obvious beta-low gamma peaks (Figures S5A,C) no signs of slow oscillations appeared in the average (Figures S5B,D). Given that we have bandpass filtered between the 20 and 45 Hz range, the central averaged peak results from the signal peak alignment whose amplitude is accumulated. The lack of underlying alpha rhythm in the average is less obvious despite the clear spectral peak in the alpha range (Figure S2A), the alpha phase to beta-low gamma CFC (Figure 1B), and the robust alpha peak-triggered average (Figures S4D,I).

Summarizing, event-related oscillatory averages did not allow us to demonstrate clear CFC. This result suggested that the coherence between the alpha band and the beta-low gamma amplitude envelope might be a spurious correlation due to the non-sinusoidal properties of the alpha rhythm.

### Occipital alpha phase to beta-low gamma power coupling during working memory delay was not modulated by LZP

So far, we have focused the analysis on the resting state conditions but it remains to be tested whether similar CFC and drug modulations can be observed under complex cognitive operations. To explore whether the amplitude of high frequency oscillations was phase-locked to the phase of slow rhythms during the WM delay periods, we computed the same CFC measures when participants engaged in a visuo-spatial WM task (Figure S1A). Given previous evidence (Lozano-Soldevilla et al., 2014), the analysis was focused on the WM delay interval (0.5–1.5 s) using the same parameters as done in the resting state analysis.

The strongest coherence appeared in the occipital beta range (16–22 Hz) with very weak modulation in the beta-low gamma band (25–45 Hz; Figures S1B–D). LZP did not modulate the coupling significantly in any frequency range (cluster-based nonparametric permutation test, *p* > 0.16). Magnitude squared bicoherence showed the strongest coupling in the first two harmonics over occipital sensors (8–12 Hz; 18–22 Hz) but they were not significantly modulated by LZP (cluster-based nonparametric permutation test, *p* > 0.11; Figure S6). CFD did not show any significant directionality coupling (Figure S7) and LZP did not modulate it.

In conclusion, we observed CFCoh between alpha phase and beta amplitude during the WM delay maintenance period. We found occipital phase-to-phase coherence between alpha and its first two harmonics. CFD did not show convincing evidence of directionality within the frequency range under study. Lastly, the pharmacological GABAergic enhancement did not significantly change any of the CFC metrics used here.

### Signal morphology produced spurious CFCoh and CFD

**Figure 3:**
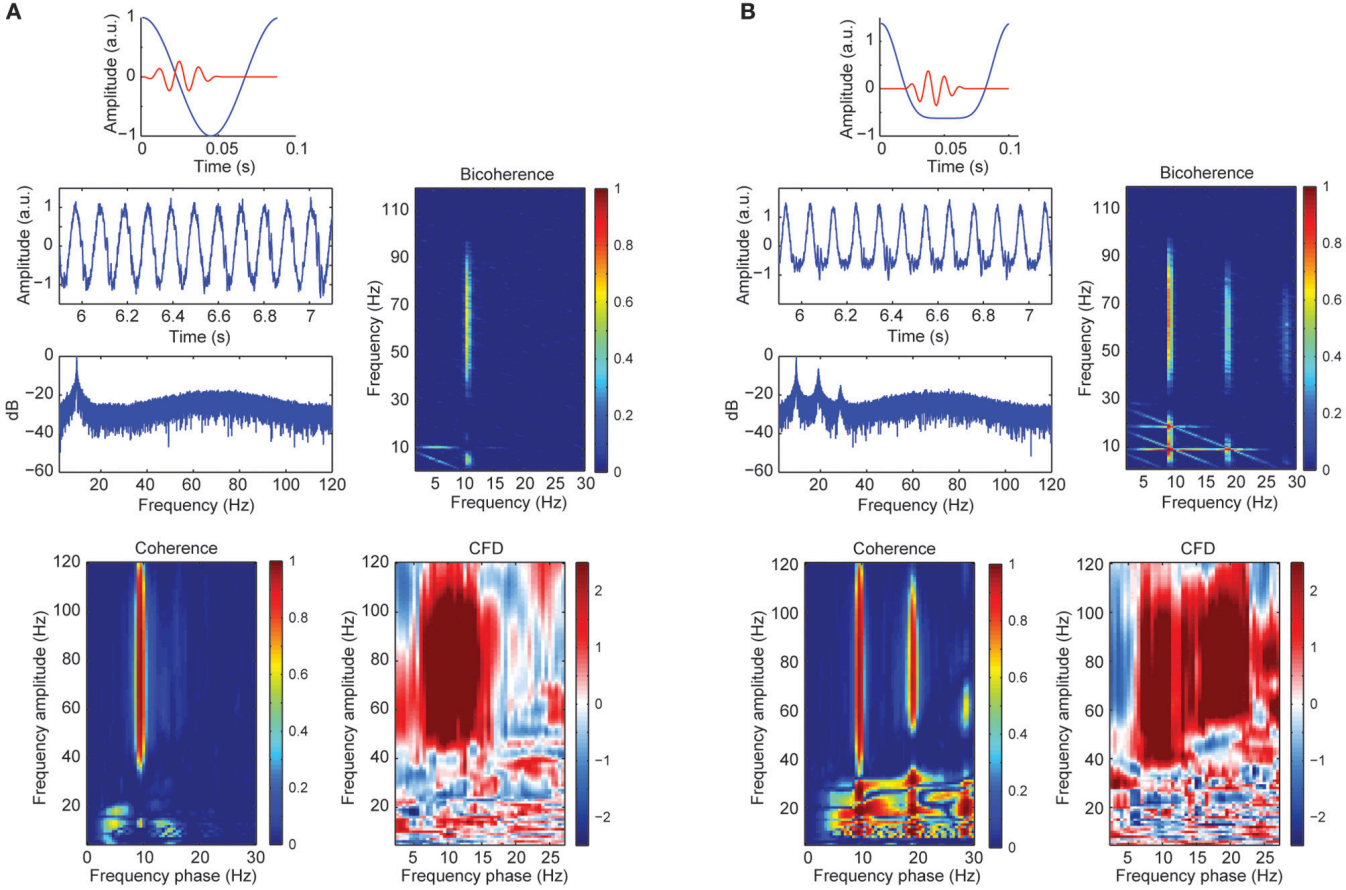
**(A)** Simulated alpha sinusoid (~10 Hz) and gamma band (60–80 Hz) CFC. Top: in blue full cosine cycle of a ~10 Hz signal coupled to a gamma 50 ms burst (red). Middle left: time course of the alpha-gamma coupled signal with random noise and the power spectrum in decibels (dB). Middle right: bicoherence of the simulated coupled signal. Color code represents the magnitude squared coherence between a pair of frequencies (x and y axes) and its sum. Bottom left: CFCoh of the simulated signal. Color code represents magnitude squared coherence between y-axes amplitude envelope frequencies and the phase of the raw signal (x-axes). Bottom right: CFD between high frequencies (y-axes) and low frequencies (x-axes). Color code indicates the normalized $\tilde{\Psi }$ weighted average of the CFD. **(B)** Simulated alpha signal with non-sinusoidal shape (~10 Hz) coupled to the amplitude of the gamma band (60–80 Hz). Top: in blue one asymmetric cycle on a ~10 Hz signal coupled to a gamma 50 ms burst (red). Same figure conventions as in **(A)**. a.u., Arbitrary units.

A sizable part of our empirical CFC patters might be explained by harmonics from the alpha band due to its well-known non-sinusoidal wave morphology properties. To test this possibility, we created synthetic data with a variety of wave morphologies to study systematically its influence on CFC metrics. Figure 3A showed CFC between narrow gamma band amplitude (60–80 Hz) and alpha phase (8–12 Hz) as simulated in case 1. In addition, we observed a phase-phase coupling using bicoherence, with no contributions in at the harmonics of the alpha wave. In addition, we observed a robust CFD, consistent with the simulated signal where the gamma bursts were leading the alpha cycles (between 0 and 20°C of the phase of the slow frequency). The observed broadband coherence and directionality extended well beyond the simulated parameters (60–80 Hz), presumably due to spectral leakage. Figure 3B represented the simulated signal from case 2 with a non-sinusoidal morphology of the alpha signal: higher magnitude of the amplitude of the peaks compared to the troughs. The power spectrum revealed three prominent peaks around ~10 Hz (fundamental frequency), ~20 and ~30 Hz (harmonic frequencies). As a consequence of the harmonics, CFCoh and bicoherence not only showed the high gamma to alpha (fundamental component ~10 Hz; x-axes CFCoh comodulogram Figure 3B) coupling but also CFC at the harmonic frequencies. Interestingly we found CFD not only to be robust between high gamma and the ~10 Hz phase but also with the ~20 Hz phase, similar as in the CFCoh. This can be explained because the harmonics were, by definition, phase-coupled to the fundamental frequency and therefore the high gamma coupled to all three components. Strikingly, no CFD was found at the lower frequencies (10–30 Hz), whereas the latter showed strong CFCoh.

**Figure 4:**
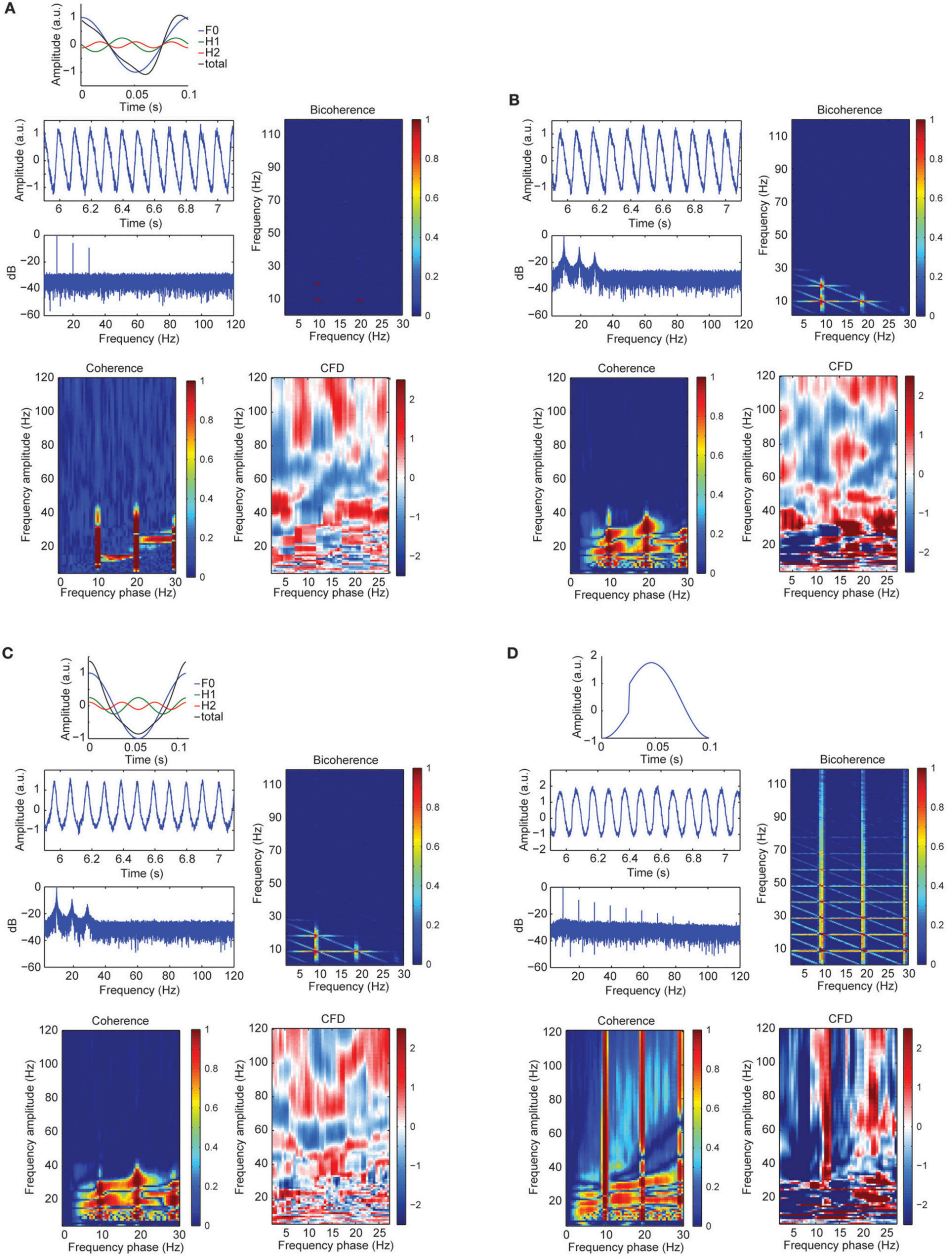
**(A)** Signal wave morphology and period fluctuations produced spurious CFC and CFD. Top: full cosine cycle of the 10 Hz (blue), 20 Hz (green), and 30 Hz (red), and the resulted signal (black) taking the sum over all the signals (F1 + H1 + H2 = total). Harmonic phase φ was set to π/2 (see simulation case 3 equation for more details). Middle left: time course of the alpha plus harmonics with random noise and the power spectrum in decibels (dB). Middle right: bicoherence of the simulated sawtooth signal. Color code represents the magnitude squared coherence between a pair of frequencies (x- and y-axes) and its sum. Bottom left: CFCoh of the simulated signal. Color code represents magnitude squared coherence between y-axes amplitude envelope frequencies and the phase of the raw signal (x-axes). Bottom right: CFD between high frequencies (y-axes) and low frequencies (x-axes). Color code indicates the normalized $\tilde{\Psi }$ weighted average of the CFD slope. **(B)** Time course of the simulated alpha sinusoid (F1 = ~10 Hz) and its first two harmonics (H1 = ~20 Hz; H2 = ~30 Hz) with period fluctuations and random noise. Same sawtooth wave as in Figure 2A top was used. Below, the power spectrum in decibels (dB). CFCoh, CFD and bicoherence have the same figure conventions as in Figure 2A. (C) Simulated alpha sinusoid (F1 = ~10 Hz) and its first two harmonics with period fluctuations. Top: full cosine cycle of the ~10 Hz (blue), ~20 Hz (green), and ~30 Hz (red) and the resulted signal (black) taking the sum over all the signals (F1 + H1 + H2 = total). Harmonic phase φ was set to 2π (see simulation case 3) equation for more details). Same figure conventions as in **(A)**. **(D)** Simulated imperfect sinusoid (see simulation case 4). Top: full cosine cycle of 10 Hz with sharp edges and no period fluctuations. Same figure conventions as in **(A)**.

To further assess the sensitivity of CFC metrics to signal morphology, in simulation case 3 we constructed a sawtooth signal with a fundamental and two harmonics, varying the signal morphology and keeping the frequency constant (i.e., no period fluctuations). Figure 4A showed a curved negative sawtooth with very clean spectral peaks at the alpha frequency and its first two harmonics. We found that CFCoh and bicoherence captured the spurious coupling between frequencies, whereas CFD showed low values not exceeding the significance threshold. Non-surprisingly, adding frequency fluctuations to the same sawtooth simulation yielded significant CFD (Figure 4B).

**Figure 5:**
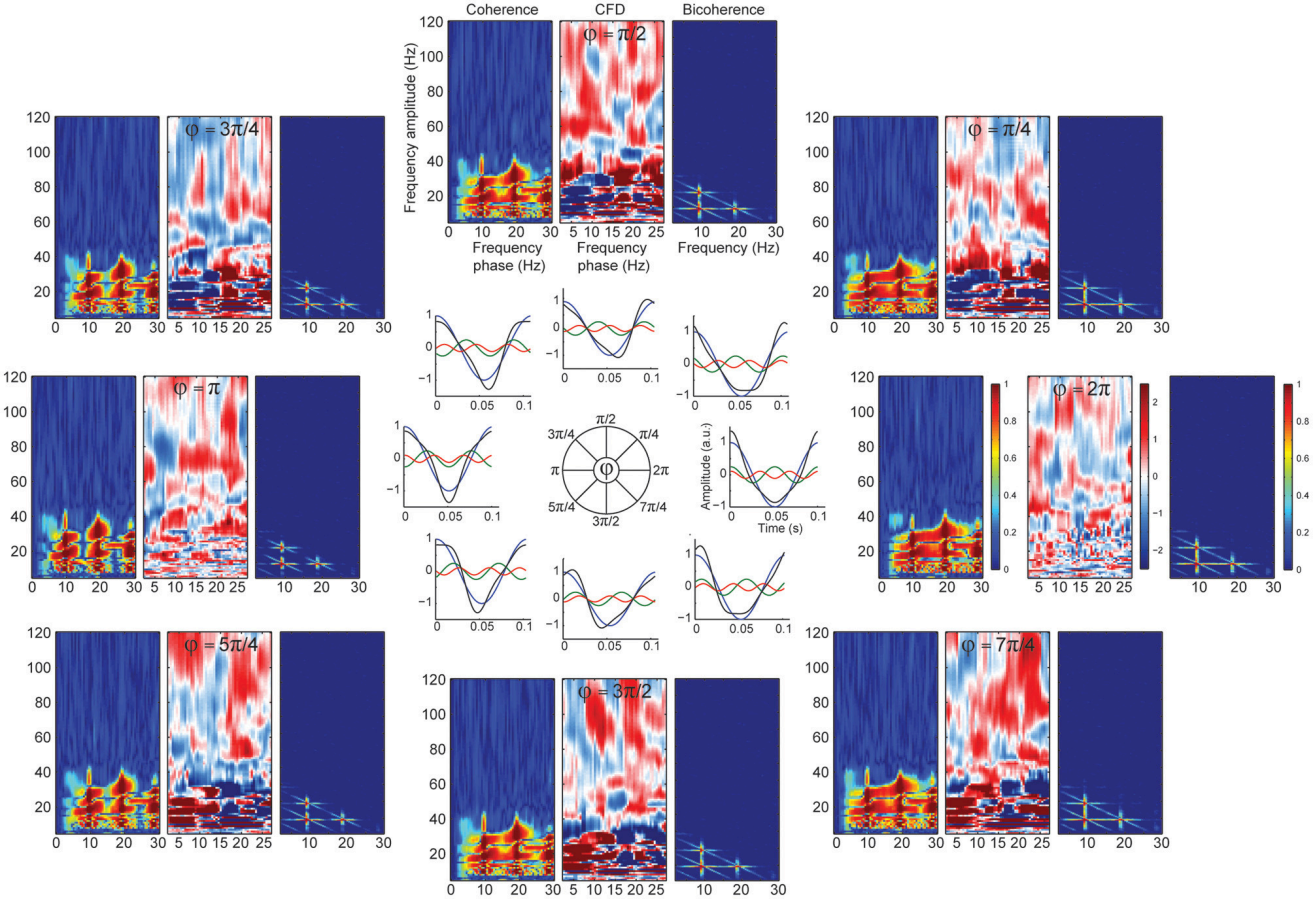
**CFD index changed as a function of wave morphology**. Simulated alpha sinusoid (F1 = ~10 Hz) and its first two harmonics (H1 = ~20 Hz; H2 = ~30 Hz) with period fluctuations. Inner circle: full cosine cycles of the ~10 Hz (blue), ~20 Hz (green) and ~30 Hz (red) and the resulted signal (black) taking the sum over all the signals (F1 + H1 + H2 = total). Harmonic phase φ was set to the different phases as indicated in the circle (see simulation case 3 equation for more details) organized in a clock-wise manner. Each time-resolved single cycle was associated to CFCoh, CFD, and bicoherence plots. Figure scales and conventions were the same as in Figure 1.

Significant CFC could be observed in the absence of CFD if the amplitude envelope of the higher frequencies and the analytic phase of the slow frequencies covary in time without phase delays (Jiang et al., 2015). Some type of periodic non-sinusoidal wave morphologies might be unable to detect significant CFD due to the consistent zero phase-lag CFC between the fundamental frequency and its harmonics. To test this possibility, we made a variant of case 3 in which the harmonics were aligned to the fundamental frequency, showing non-sinusoidal shape properties without phase lead, nor lag (Figure 4C). As predicted, we observed spurious CFC between alpha and its harmonics but no significant directionality within the harmonic frequency ranges. To explore whether CFD could be insensitive to spurious CFC due to the non-sinusoidal properties of an alpha-like signal, we systematically modulated the φ-value to simulate a variety of waveforms that are displayed in Figure 5 (see simulation case 3 for details). We found two cases on where CFD between alpha-like non-sinusoidal synthetic signal and its harmonics: the phase φ being π or 2π. In both cases the signal had a non-sinusoidal wave morphology (π, troughs with more amplitude than peaks; 2π, peaks more amplitude than troughs) and near zero phase-lag between the amplitude envelope of the harmonics and the phase of the fundamental frequency. Among other φ values, CFD changed as a function of the sawtooth peak: negative CFD was obtained with a negative sawtooth waveforms and positive CFD was obtained with a positive sawtooth waveforms. Comparing the upper (at 12:00) and lower (at 6:00) panels in Figure 5 one can observe the sign of the directionality being flipped. In conclusion, we identified for the first time the wave morphologies that produced spurious CFCoh and CFD.

### Sharp edges in the signal produce spurious CFD

Although CFD should not yield significant directionality when the frequency of a periodic signal does not fluctuate, sharp edge transients could still introduce spurious directionality. To assess the influence of sharp edge artifacts on the CFD index, in simulation case 4 we replicated the example described in Kramer et al. (2008). As seen in Figure 4D, we observed not only strong CFCoh between the fundamental frequency and multiple harmonics, but also robust CFD between multiple frequencies. Therefore, we found that CFD yields multiple false positives when oscillations contain edge artifacts (Jiang et al., 2015). This is true even when the periodicity of the signal did not fluctuate.

## Discussion

Here, we analyzed a pharmaco-MEG dataset to investigate how CFC metrics were modulated by LZP during rest and WM maintenance. During rest, we demonstrated that while occipital alpha power decreased with LZP (Link et al., 1991; Schreckenberger et al., 2004; Ahveninen et al., 2007), sensorimotor beta power increased with drug dosage (Baker and Baker, 2003; Jensen et al., 2005; Hall et al., 2010). We found alpha phase to beta-low gamma amplitude coupling over occipital sensors that decreased with 1.5 mg LZP during EC. However, CFD and peak-triggered averages did not provide additional phase-to-amplitude coupling evidence. In addition, we found phase-to-phase coupling between alpha and beta over occipital sensors. The strongest CFCoh we observed during WM conditions was between alpha phase ~10 Hz and beta amplitude ~20 Hz, with robust phase-to-phase alpha-beta coupling without significant drug modulations.

### Cross-frequency coupling between alpha and beta-low gamma

Several MEG studies have investigated on the role of phase-to-amplitude coupling under rest and task conditions. In one of the first attempts, Osipova et al. (2008) found that the ongoing gamma (30–70 Hz) power was phase-locked to the alpha band (8–12 Hz) over posterior regions under resting state. This was mainly observed in subjects with strong alpha power. We however found a coupling in lower frequency range over occipital sensors in a bigger sample. A similar gamma frequency range (19–76 Hz) phase-locked to slower alpha (5–13 Hz) was reported by Hawellek et al. (2013). A very recent study found that the phase of spontaneous alpha oscillations in thalamus coupled to ~40 Hz gamma power in parieto-occipital cortex during EC conditions (Roux et al., 2013). ECoC recordings in humans reported the same phenomenon both in lower (Fitzgerald et al., 2013) and higher gamma band frequency during resting state (Bahramisharif et al., 2013). In summary, the absence of high frequency gamma power in our MEG data could be related to due to a lower signal-to-noise ratio for MEG as compared to ECoG data.

### Neurophysiological oscillations with non-sinusoidal wave morphologies produce spurious CFC

The results of the simulations presented here raised a challenge to CFC analyses of signals whose waveform is known to be non-sinusoidal, such as the occipital alpha (Stam et al., 1999; Nikulin et al., 2007), hippocampal theta (Belluscio et al., 2012), and the sensorimotor mu-rhythm (Salmelin and Hari, 1994). The latter is in fact quite revealing, given that it was originally called “*rhythme en arceau”* by Gastaut (1952) due to its arch-like shape.

Our synthetic data showed convincingly that periodic signals deviating from sines and cosines (i.e., the filter kernel) produced harmonics in the power spectrum and these harmonics were, by definition, phase-to-amplitude coupled (Figure 1). Taken this fact into account, we cannot claim with absolute certainty that all CFCoh patterns we observed in our empirical data (Figure 1) were produced by the non-sinusoidal properties of the wave morphology of the alpha rhythm. However, it is undisputable that a sizable part of our CFCoh was explained by harmonics generated by the non-sinusoidal wave morphology properties of the alpha rhythm.

The non-sinusoidal shape properties of the alpha band (i.e., magnitude of the peaks higher relative to the magnitude of the troughs) should not be confused with the alpha asymmetric amplitude fluctuation properties defined by Mazaheri and Jensen (2008). The asymmetric amplitude fluctuations were defined as the normalized ratio of the *variance* (not the amplitude) of the peaks of an oscillatory activity minus the *variance* of the troughs (Mazaheri and Jensen, 2008). The authors used a synthetic signal with the form:

(11) $$\begin{array}{l} a=A\left(t\right)*\left(1+sin\left(2\pi ft\right)\right) \end{array}$$

where *A*(*t*) is a slow signal (i.e., 3 Hz) that determined the amplitude modulation of the alpha frequency *f*. Note that, by construction, this synthetic signal will not produce harmonics because it consisted of a DC offset term and a single sine wave. This type of model might not apply to the empirical data presented here because we observed strong coupling between 10 Hz and its first two harmonics using bicoherence (Figure 2; Figure S6).

Summarizing, there are physiological reasons to believe that some neurophysiological signals have non-sinusoidal properties (i.e., waveforms deviating from sines and cosines). For instance, when assessing the coupling between different frequencies, the potential harmonic contribution to CFC should be taken into account using bicoherence or any other metric sensitive to harmonic.

### Non-zero CFC was produced by a mismatch between filter kernel and signal morphology

The results presented here were obtained using only a limited number of CFC metrics that were based on Fourier methods. We expect other methods based on band-pass filtering and Hilbert transform to yield, in principle, similar results (Canolty et al., 2006; Cohen, 2008; Penny et al., 2008; Tort et al., 2010). The reason is because there is an equivalence between wavelet, Fourier and Hilbert methods on amplitude spectral estimation (Bruns, 2004). More importantly, Lepage et al. (2013) demonstrated that the phase variance and bias of the three estimators “*are shown, in simulation and in theory, to depend on the extent to which signal present in the data is matched to the phase estimator”* (p. 915). While wavelet phase estimator outperformed Fourier and Hilbert transforms when the signal to be analyzed had a broadband pulse-like wave morphology, the Fourier approach yielded more accurate phase estimates in comparison with the other two methods when the signal to be analyzed showed a stationary sinusoidal-like wave morphology (see Lepage et al., 2013 for details). Spectral analysis of periodic signals whose wave morphologies systematically mismatch a given filter kernel (i.e., wavelet, sine/cosine) will produce harmonics, and in consequence, spurious CFC (Lozano-Soldevilla, 2015). This observation has been recently replicated by various independent research groups (Cole et al., 2016; Gerber et al., 2016; Jones, 2016; Scheffer-Teixeira and Tort, 2016).

Following this rationale, if the filter kernel can be adapted to the signal under analysis, the spectral estimation will be more accurate. Future CFC metrics could use filter kernels adapted to non-sinusoidal signals to avoid spurious correlations between frequency components.

### Cross-frequency directionality index produced spurious directionality

Present simulations and empirical results showed that spurious CFCoh and CFD may be indeed found between the phase of the fundamental frequency of slow oscillations and the amplitude envelope of its harmonics. Periodic signals with sawtooth-like wave morphologies produce spurious CFD. The CFD proposed by Jiang et al. (2015) is the PSI applied to the cross-frequency domain. PSI has been shown to be resistant to false positives when even when the time series to be assessed contained non-linear interactions (Nolte et al., 2010). The PSI computes directionality by comparing two independent time series, but the CFD was computed here in the same recording (one time series) between different frequency components. The simulations presented here clearly demonstrated that spurious coupling (CFCoh and CFD) between the fundamental frequency and its harmonics can also be narrowband (20–45 Hz). Our synthetic data revealed for the first time that the CFD metric gave false positives not only when the signal contain sharp edge artifacts (Kramer et al., 2008) but also with signal whose wave morphology was sawtooth-like. We want to stress that this is especially problematic if the CFC occurs over the first harmonics as in our case. We recommend future CFD users to check the low frequency signal morphology and other potential sources of non-linear coupling that could produce spurious results (Aru et al., 2015).

One way to reduce this problem would consist in computing CFCoh and CFD between sets of independent recordings (Maris et al., 2011; van der Meij et al., 2012, 2015; Aru et al., 2015). This approach presumably attenuatates contribution of volume conduction that could potentially spread non-sinusoidal periodic signals that could yield spurious correlations. For example, Spaak et al. found stronger CFC between layers in monkey V1 (alpha phase of infragranular layer coupled to gamma power of supragranular layer) in comparison with CFC within the infragranular layer (Spaak et al., 2012). However, a recent study found that the infragranular alpha sources might reflected a mix of local activity of field potentials (LFP) and contaminated activity that was volume-conduced from supragranular layers (Haegens et al., 2015). This adds more complexity not only to the interpretation of LFP and current source density results but also to the usefulness of the CFC in cases on where the cortical connectome unambiguously determined.

Datasets including spikes and LFP could help to dissociate the gamma oscillations from the potential harmonics from non-sinusoidal properties of slower rhythms. In this direction, Colgin et al. (2009) found that both fast and slow gamma oscillations in different circuitry of rat hippocampus locked to spikes at different phases. Specifically, this would mean that both slow and fast gamma are rhythms not only related to spiking activity between regions but also modulated by the phase of the ongoing theta activity. However, this approach is not exempt of problems. It is not easy to establish unambiguously which variables are synchronized using different coherence measures (see Buzsáki and Schomburg, 2015 for an elaborated argumentation). Gamma power can be contaminated by the spike activity, making very challenging to disentangle independent oscillatory gamma estimates from the spike activity (Buzsáki and Schomburg, 2015; Ray and Maunsell, 2015). This is a similar problem described here, but this time the harmonic frequencies are originated from the non-sinusoidal properties of the spike wave morphology. Indeed, an empirical study found that the contribution of these spike-related harmonics can go as low as 10 Hz (Waldert et al., 2013). Although spiking activity can definitely help to understand LFP activity, its interpretation in relation to neuronal oscillations needs to be done carefully.

## Conclusion

In conclusion, our findings showed CFC between spontaneous alpha and beta-low gamma over occipital sensors during rest and WM. However, the observed phase-to-amplitude coupling contained harmonic power contributions presumably from the non-sinusoidal properties of the alpha wave morphology. Although alpha harmonics may not fully explain the whole CFC pattern observed here, this seems to be the most parsimonious interpretation. In a broader scope, the present results highlighted the fact that periodic signals with non-sinusoidal wave morphology generates non-zero CFC and spurious CFD (i.e., sawtooth-like morphologies). We suggest a cautionary interpretation of CFC results based on current models that assume that communication between neuronal ensembles are implemented as cross-frequency interactions between gamma and alpha activity (Jensen and Mazaheri, 2010).

## Author contributions

DL designed the study, collected and analyzed the data. DL, Nt, and RO discussed the results and wrote the paper.

### Conflict of interest statement

The authors declare that the research was conducted in the absence of any commercial or financial relationships that could be construed as a potential conflict of interest.

## Supplementary material

Figure S1 **LZP did not modulate alpha-phase to beta-low gamma amplitude coupling during WM delay. (A)** Delayed match to sample WM task. Participants had to keep fixations while covertly attend to the cued visual hemifield to encode the target items. The task consisted to compare the cued items, compare them with a probe and decide whether they matched or not. **(B)** Grand mean topographic representation of alpha-phase to beta (16–22 Hz; left) and to beta-low gamma amplitude coupling (25–45 Hz; right) during WM delay (0.5 to 1.5 s). Color code represents magnitude squared coherence. Note that topographies have different scale. The strongest coherence values appeared over occipito-parietal sensors. Grand mean frequency-by-frequency coherence comodulogram (middle) of the occipital axial gradiometers of interest as in Figure S2A. Black rectangles indicate the x-y frequency selections used in the statistical analysis and topographic representations (8–12 Hz; 16–22 Hz; 25–45 Hz). Comodulograms have the same coherence scale as alpha—beta topography (left). **(C,D)** are the same as **(B)** but for 0.5 and 1.5 mg LZP, respectively.

Figure S2 **LZP modulations in the alpha (8–12 Hz) and beta (15–30 Hz) bands during rest. (A)** Grand mean topographic representation of alpha power during EO (left) and EC (right). Color code represents relative power changes with respect to the mean of the power spectrum (5–40 Hz). The rows reflect the drug levels: Placebo, 0.5 and 1.5 mg LZP. Sensors marked in bold display statistical significant drug main effect (repeated-measures ANOVA) using a cluster-based nonparametric permutation test. **(B)** Grand average of power spectra [sensors marked white in **(A)**] estimated for the EO (top) and EC (bottom) and all drug sessions. Inserts show a repeated-measures ANOVA confirming that the occipital alpha power decreased with LZP. Error bars show the SEM. **(C)** Occipito-parietal sources reflected the alpha power decrease under 1.5 mg LZP dosage relative to placebo (EO and EC averaged). **(D)** Gran mean topographic representation of beta power during EO (left) and EC (right) during rest [same conventions as in **(A)**]. **(E)** Grand average of power spectra [sensors marked white **(D)**] estimated for the EO (top) and EC (bottom) and all drug sessions. Inserts showed a repeated-measures ANOVA confirming that the sensorimotor beta power increased with LZP dosage; same conventions as in Figure 1B. Error bars show the SEM. **(F)** The sources in the sensorimotor cortex reflected the beta power increase under 1.5 mg LZP dosage relative to placebo (EO and EC averaged) [same conventions as in **(C)**]. The peak of the beta source lied out of the brain due to inaccuracies after the individual head model normalization to the MNI space. ^†^*p* < 1 × 10^−6^; ^***^*p* < 1 × 10^−5^; ^**^*p* < 0.001; ^*^*p* < 0.05.

Figure S3 **LZP did not significantly change CFD during EC. (A)** Grand mean topographic representation of the CFD (8–12 Hz; 20–45 Hz) for the different drug conditions. White sensors were significantly modulated by LZP in Figure 1. The color code indicates the normalized $\tilde{\Psi }$ weighted average of the CFD slope (Ψ = $\tilde{\Psi }$*/std(*$\tilde{\Psi }$*)*). **(B)** Grand mean normalized CFD index averaged over the occipital sensors marked in topographies **(A)** for each drug session. Note the low sigma values we obtained (± ~0.3; ± 2 was considered significant). **(C)** CFD weighted average ($\tilde{\Psi }$) and **(D)** standard deviation of the slope (*std(*$\tilde{\Psi }$*)*) averaged over the occipital sensors marked in topographies for each drug session.

Figure S4 **Time-frequency representations (TFR) of induced power locked to the peaks of the alpha oscillations during rest. (A)** Grand mean TFRs from left and right hemisphere MEG axial gradiometers [for white sensors in **(E)**] time-locked to the alpha peaks (*t* = 0 s) under placebo session during EO. TFRs were calculated for each single trial and then averaged. Color code represents the relative power normalized to the average power per frequency (y-axes). **(B)** TFRs of induced power locked to the peak of alpha oscillations for 0.5 mg LZP during EO. Same conventions as in **(A)**. **(C)** TFRs of induced power locked to the peak of alpha oscillations during 1.5 mg LZP during EO. Same conventions as in **(A)**. **(D)** Grand mean of event-related fields locked to the alpha peaks. The peaks were found after bandpass filtering of the data (8–12 Hz). MEG axial gradiometers from left and right hemispheres (marked white in topography) during EO for all drug levels. **(E)** Grand mean topographic representation of alpha peak-triggered average over the period (–0.015 to 0.015 s) around the alpha peak (*t* = 0 s). Marked sensors indicate statistical significant main effect (repeated-measures ANOVA) between treatments using the cluster-based nonparametric permutation test. **(F–J)** are the same as **(A–E)** but for EC respectively.

Figure S5 **Beta-low gamma peak-triggered average during rest. (A)** Grand mean of event-related fields locked to the beta-low gamma (20–45 Hz) peaks. MEG axial gradiometers from left and right hemispheres [marked white in **(B)**] during EO for all drug sessions. Vertical dashed lines at ± 0.05 s indicates the lower limit of the bandpass filter used for peak detection (1/0.05 s = 20 Hz). Beyond the ± 0.05 s limit, the underlying alpha oscillations were highly reduced if any. **(B)** Grand mean topographic representation of beta-low gamma peak-triggered average taken around the peak period (–0.015 to 0.015 s). Marked bold sensors of interest based on Figure S2A. Panels **(C,D)** are the same as **(A,B)** but for EC, respectively with same figure conventions.

Figure S6 **LZP did not modulate bicoherence values during WM delay. (A)** Grand mean topographic representation of alpha–alpha (8–12 Hz; 8–12 Hz; left) and alpha–beta coupling (8–12 Hz; 18–22 Hz; right) during WM delay (0.5–1.5 s). Color code represents magnitude-squared bicoherence. Note that the topographic plots have different scale. The strongest bicoherence values appeared over occipito-parietal sensors. Grand mean frequency-by-frequency bicoherence comodulogram (middle) of occipital axial gradiometers of interest chosen from Figure S2A. Black rectangles indicate the x-y frequency selections used in the statistical analysis and topographic representations (8–12 Hz; 8–12 Hz; 18–22 Hz). Comodulograms have the same scale as alpha–alpha topography (left). Panels **(B,C)** are the same as **(A)** but for 0.5 and 1.5 mg LZP, respectively.

Figure S7 **LZP did not change CFD during WM delay. (A)** Grand mean topographic representation of alpha-beta CFD (16–22 Hz) during WM delay for each drug separately. Color code indicates the normalized $\tilde{\Psi }$ weighted average of the CFD slope [Ψ = $\tilde{\Psi }$/*std*($\tilde{\Psi }$); color scale same as in **(B)**]. **(B)** Grand mean normalized CFD index taken over the occipital sensors of interest chosen from Figure S2A. Note the low sigma values we obtained (~ ±0.6; ±2 is considered significant). **(C)** CFD weighted average ($\tilde{\Psi }$) and **(D)** standard deviation of the slope (*std(*$\tilde{\Psi }$*)*) taken over the same occipital sensors for each drug session respectively. Drug sessions are displayed in different columns.
