# Supplementary figures and images for: Neuronal Oscillations with Non-sinusoidal Morphology Produce Spurious Phase-to-Amplitude Coupling and Directionality
Source: Front Comput Neurosci. 2016 Aug 22;10:87. doi: 10.3389/fncom.2016.00087 (PMC4992698; doi:10.3389/fncom.2016.00087)

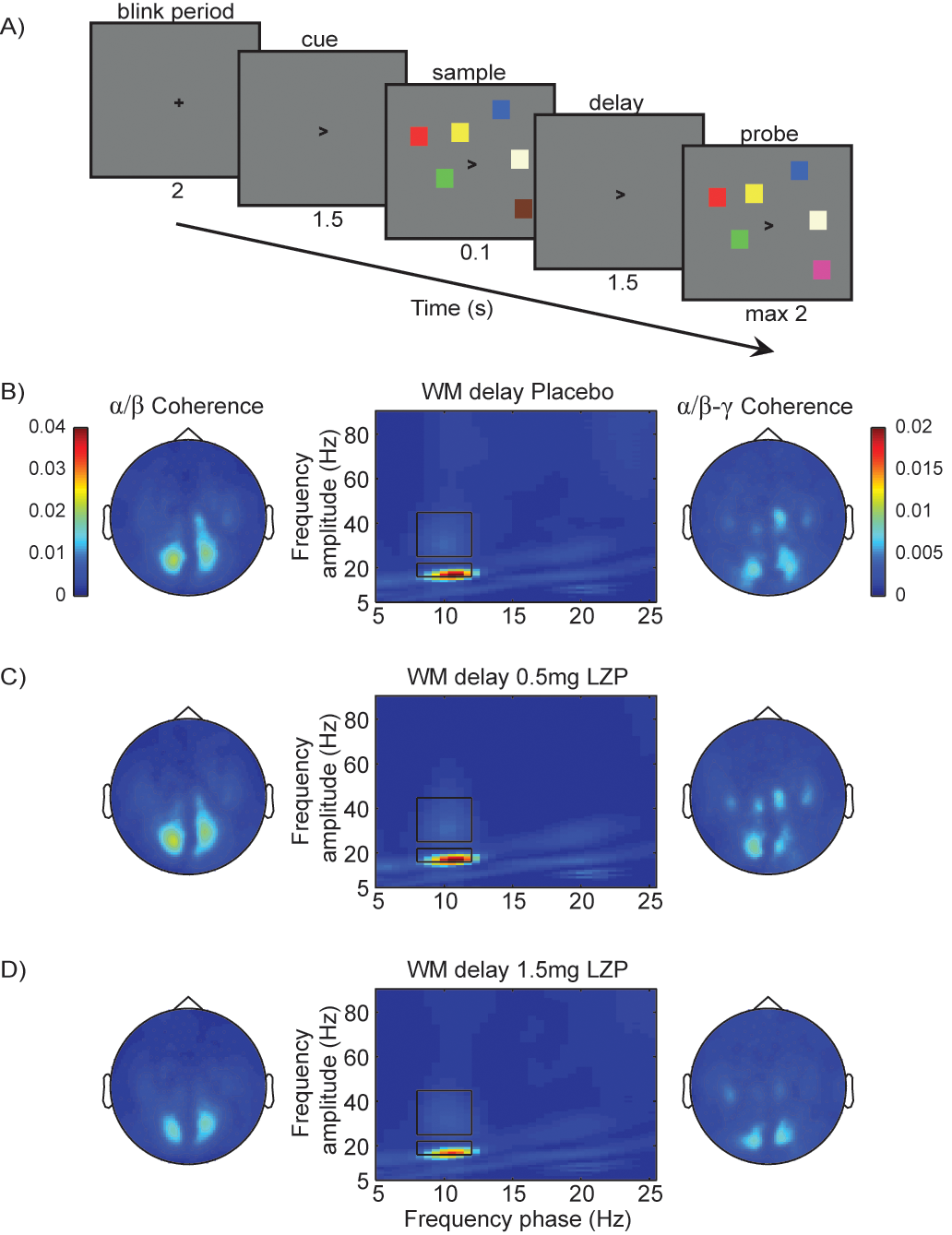

Supplement: Figure S1 — LZP did not modulate alpha-phase to beta-low gamma amplitude coupling during WM delay. (A) Delayed match to sample WM task. Participants had to keep fixations while covertly attend to the cued visual hemifield to encode the target items. The task consisted to compare the cued items, compare them with a probe and decide whether they matched or not. (B) Grand mean topographic representation of alpha-phase to beta (16–22 Hz; left) and to beta-low gamma amplitude coupling (25–45 Hz; right) during WM delay (0.5 to 1.5 s). Color code represents magnitude squared coherence. Note that topographies have different scale. The strongest coherence values appeared over occipito-parietal sensors. Grand mean frequency-by-frequency coherence comodulogram (middle) of the occipital axial gradiometers of interest as in Figure S2A. Black rectangles indicate the x-y frequency selections used in the statistical analysis and topographic representations (8–12 Hz; 16–22 Hz; 25–45 Hz). Comodulograms have the same coherence scale as alpha—beta topography (left). (C,D) are the same as (B) but for 0.5 and 1.5 mg LZP, respectively. [file Image1.TIF]

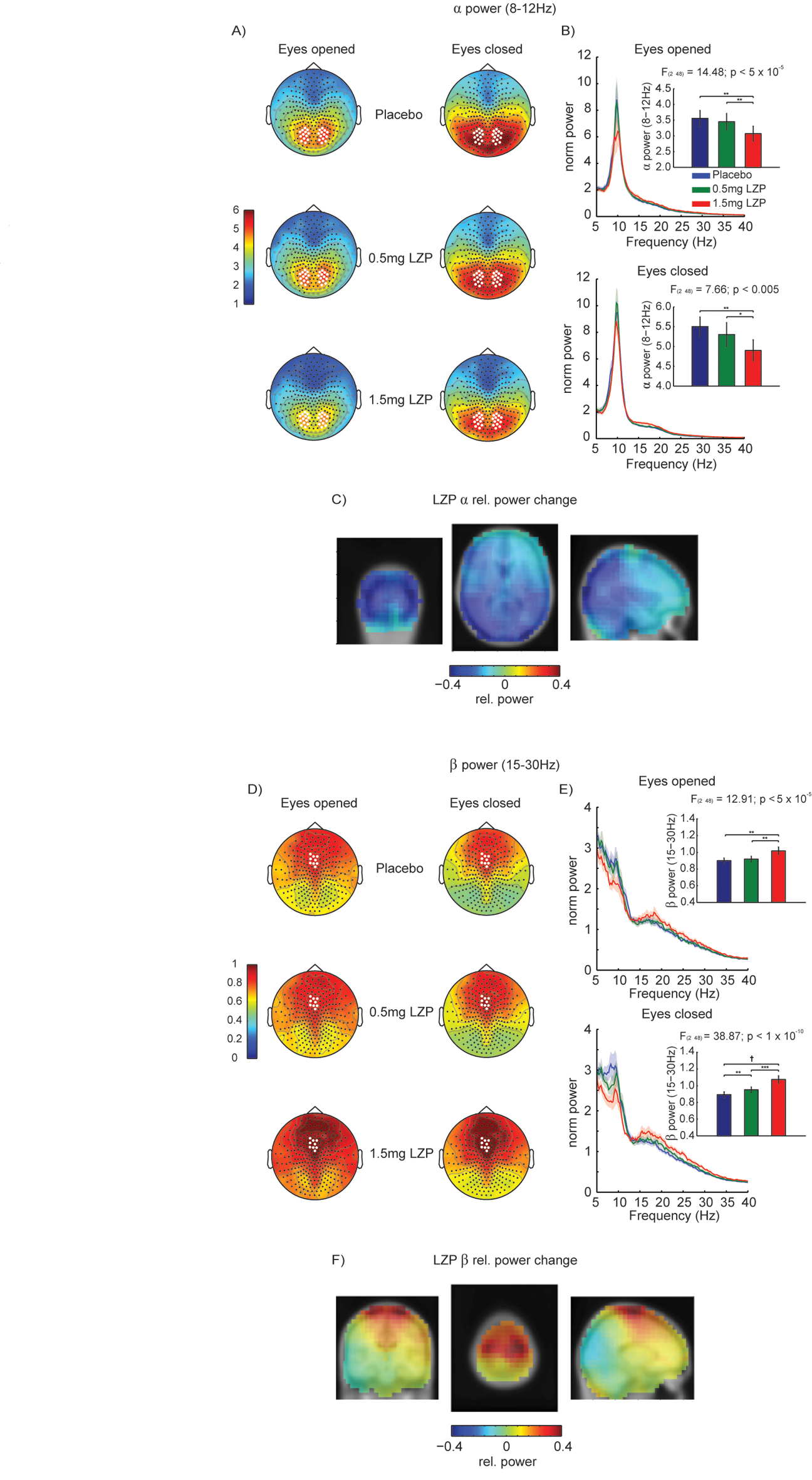

Supplement: Figure S2 — LZP modulations in the alpha (8–12 Hz) and beta (15–30 Hz) bands during rest. (A) Grand mean topographic representation of alpha power during EO (left) and EC (right). Color code represents relative power changes with respect to the mean of the power spectrum (5–40 Hz). The rows reflect the drug levels: Placebo, 0.5 and 1.5 mg LZP. Sensors marked in bold display statistical significant drug main effect (repeated-measures ANOVA) using a cluster-based nonparametric permutation test. (B) Grand average of power spectra [sensors marked white in (A)] estimated for the EO (top) and EC (bottom) and all drug sessions. Inserts show a repeated-measures ANOVA confirming that the occipital alpha power decreased with LZP. Error bars show the SEM. (C) Occipito-parietal sources reflected the alpha power decrease under 1.5 mg LZP dosage relative to placebo (EO and EC averaged). (D) Gran mean topographic representation of beta power during EO (left) and EC (right) during rest [same conventions as in (A)]. (E) Grand average of power spectra [sensors marked white (D)] estimated for the EO (top) and EC (bottom) and all drug sessions. Inserts showed a repeated-measures ANOVA confirming that the sensorimotor beta power increased with LZP dosage; same conventions as in Figure 1B. Error bars show the SEM. (F) The sources in the sensorimotor cortex reflected the beta power increase under 1.5 mg LZP dosage relative to placebo (EO and EC averaged) [same conventions as in (C)]. The peak of the beta source lied out of the brain due to inaccuracies after the individual head model normalization to the MNI space. †p < 1 × 10^−6^; ***p < 1 × 10^−5^; **p < 0.001; *p < 0.05. [file Image2.TIF]

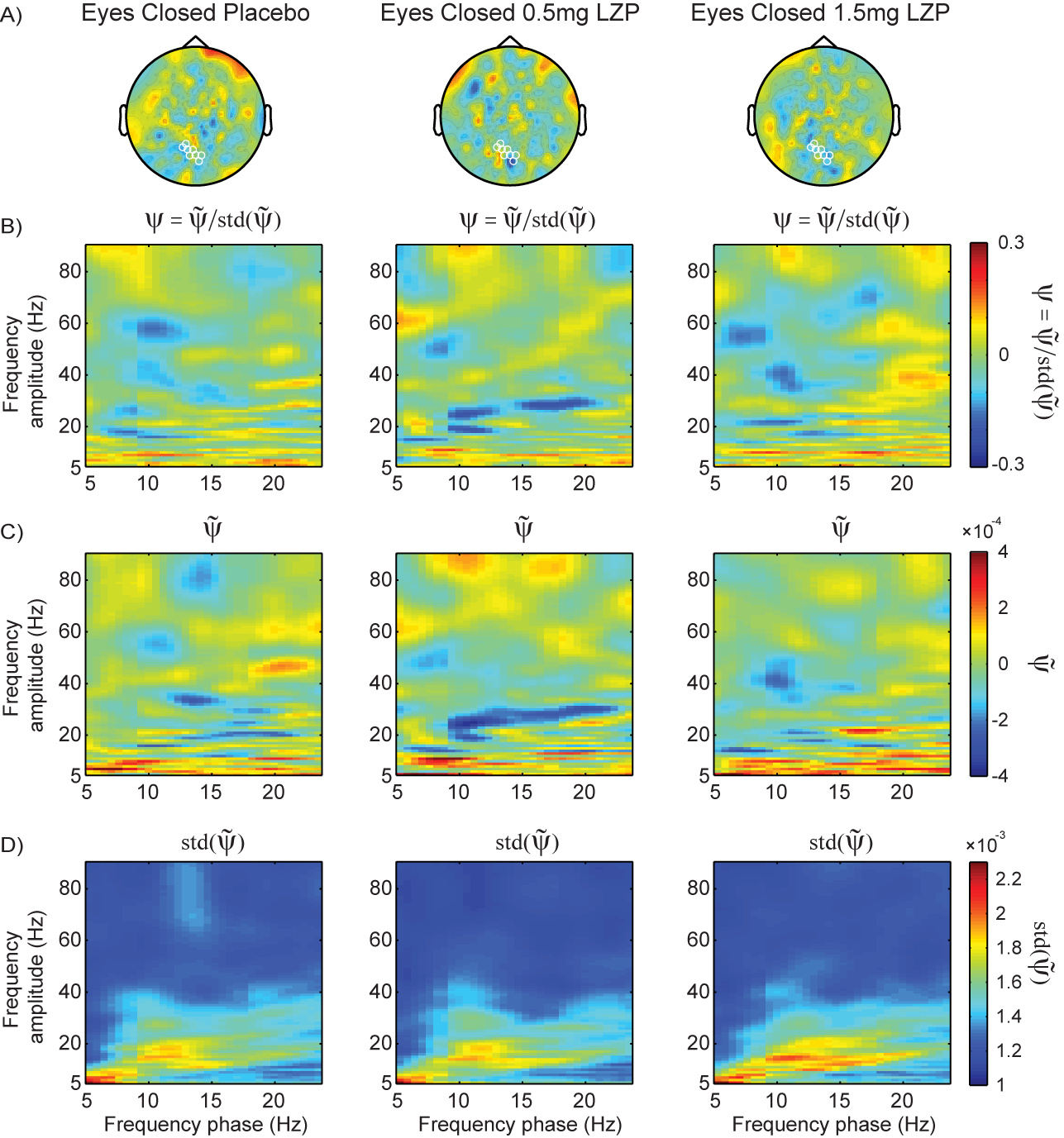

Supplement: Figure S3 — LZP did not significantly change CFD during EC. (A) Grand mean topographic representation of the CFD (8–12 Hz; 20–45 Hz) for the different drug conditions. White sensors were significantly modulated by LZP in Figure 1. The color code indicates the normalized Ψ~ weighted average of the CFD slope (Ψ = Ψ~/std(Ψ~)). (B) Grand mean normalized CFD index averaged over the occipital sensors marked in topographies (A) for each drug session. Note the low sigma values we obtained (± ~0.3; ± 2 was considered significant). (C) CFD weighted average (Ψ~) and (D) standard deviation of the slope (std(Ψ~)) averaged over the occipital sensors marked in topographies for each drug session. [file Image3.TIF]

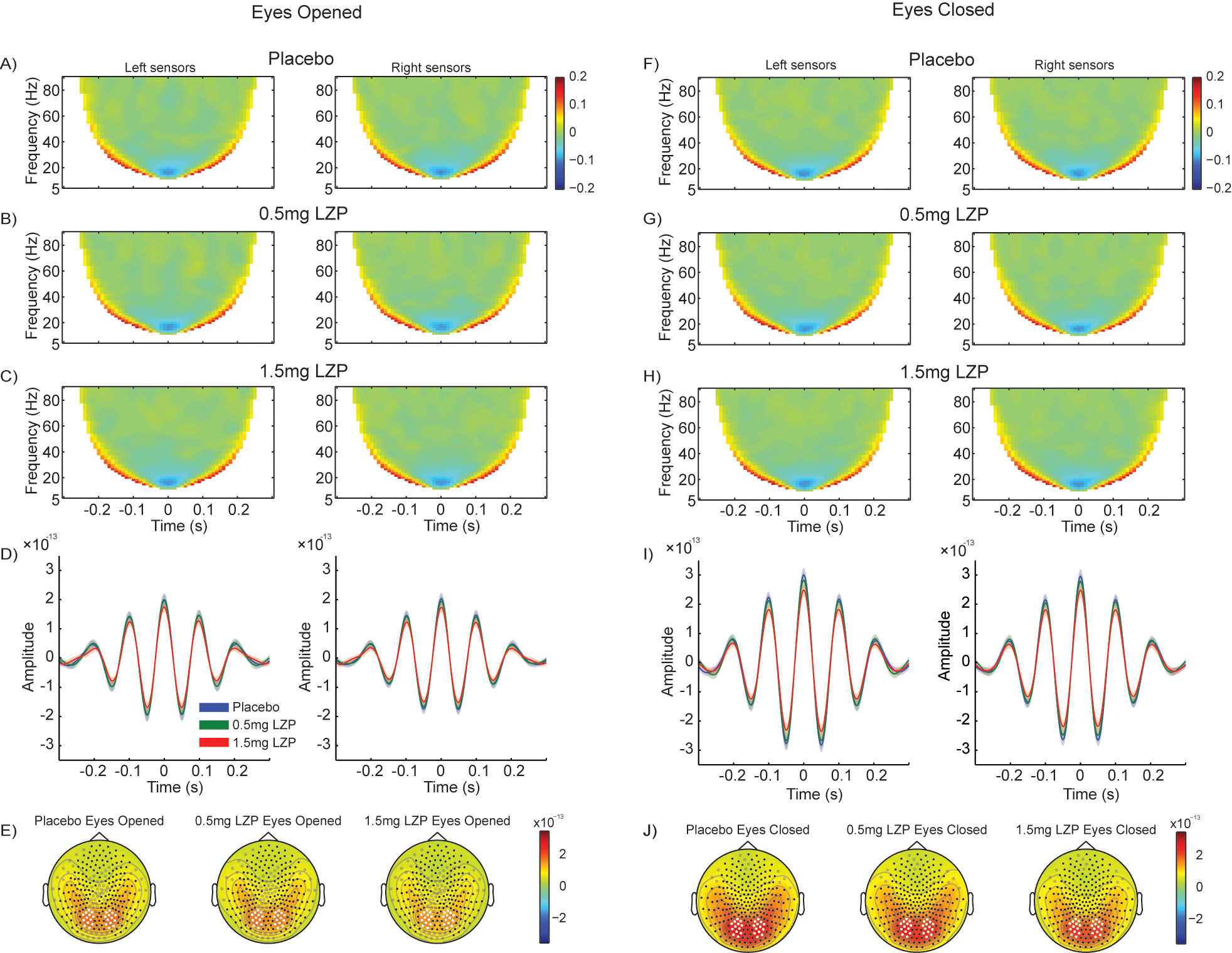

Supplement: Figure S4 — Time-frequency representations (TFR) of induced power locked to the peaks of the alpha oscillations during rest. (A) Grand mean TFRs from left and right hemisphere MEG axial gradiometers [for white sensors in (E)] time-locked to the alpha peaks (t = 0 s) under placebo session during EO. TFRs were calculated for each single trial and then averaged. Color code represents the relative power normalized to the average power per frequency (y-axes). (B) TFRs of induced power locked to the peak of alpha oscillations for 0.5 mg LZP during EO. Same conventions as in (A). (C) TFRs of induced power locked to the peak of alpha oscillations during 1.5 mg LZP during EO. Same conventions as in (A). (D) Grand mean of event-related fields locked to the alpha peaks. The peaks were found after bandpass filtering of the data (8–12 Hz). MEG axial gradiometers from left and right hemispheres (marked white in topography) during EO for all drug levels. (E) Grand mean topographic representation of alpha peak-triggered average over the period (–0.015 to 0.015 s) around the alpha peak (t = 0 s). Marked sensors indicate statistical significant main effect (repeated-measures ANOVA) between treatments using the cluster-based nonparametric permutation test. (F–J) are the same as (A–E) but for EC respectively. [file Image4.TIF]

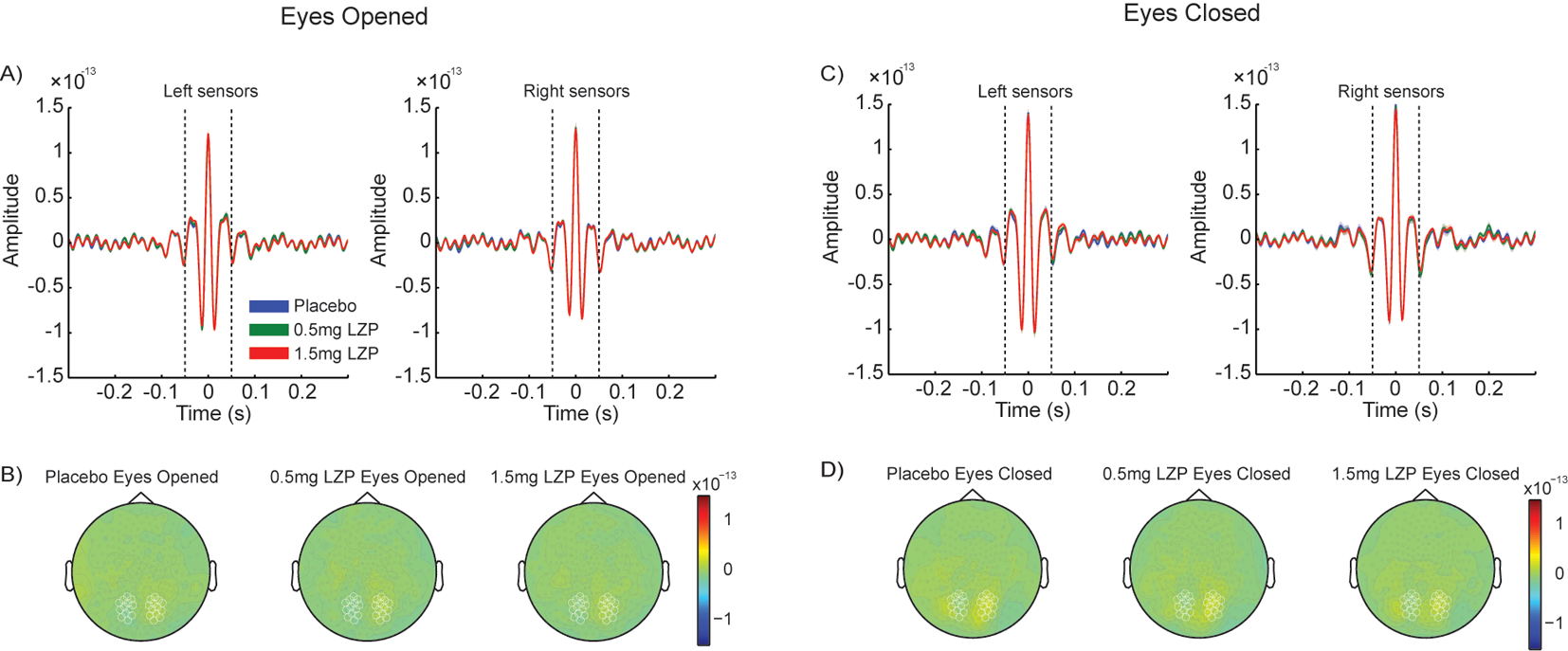

Supplement: Figure S5 — Beta-low gamma peak-triggered average during rest. (A) Grand mean of event-related fields locked to the beta-low gamma (20–45 Hz) peaks. MEG axial gradiometers from left and right hemispheres [marked white in (B)] during EO for all drug sessions. Vertical dashed lines at ± 0.05 s indicates the lower limit of the bandpass filter used for peak detection (1/0.05 s = 20 Hz). Beyond the ± 0.05 s limit, the underlying alpha oscillations were highly reduced if any. (B) Grand mean topographic representation of beta-low gamma peak-triggered average taken around the peak period (–0.015 to 0.015 s). Marked bold sensors of interest based on Figure S2A. Panels (C,D) are the same as (A,B) but for EC, respectively with same figure conventions. [file Image5.TIF]

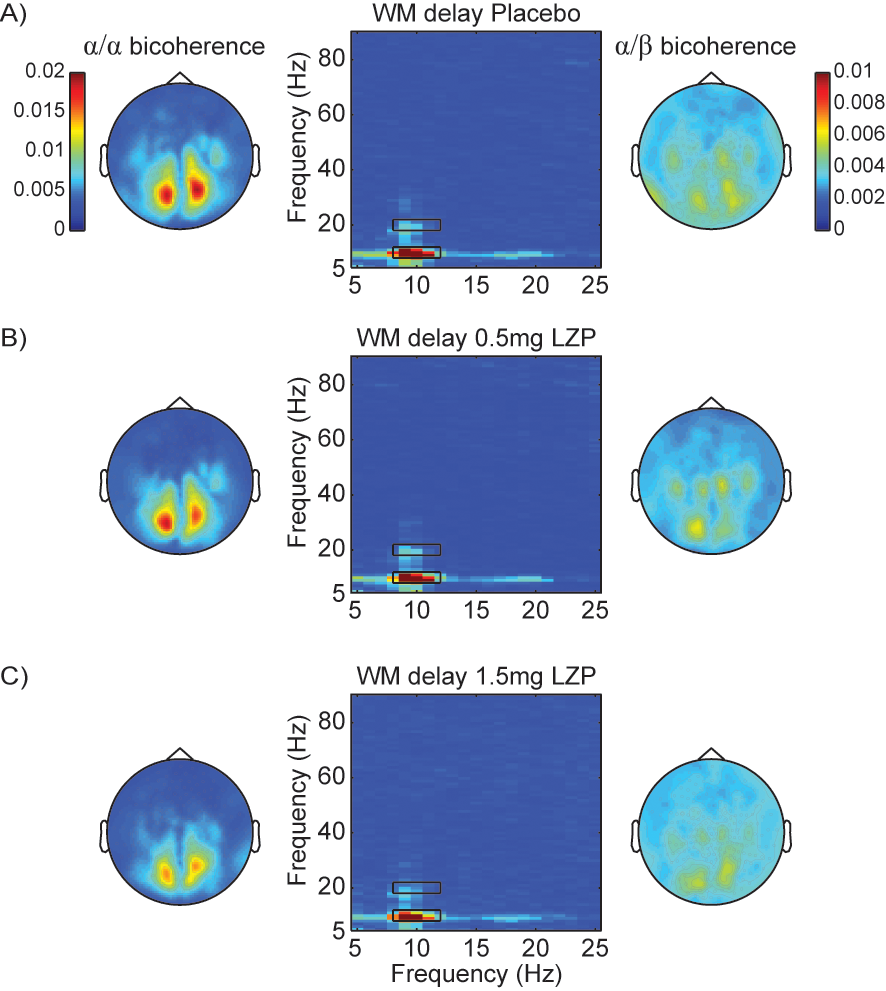

Supplement: Figure S6 — LZP did not modulate bicoherence values during WM delay. (A) Grand mean topographic representation of alpha–alpha (8–12 Hz; 8–12 Hz; left) and alpha–beta coupling (8–12 Hz; 18–22 Hz; right) during WM delay (0.5–1.5 s). Color code represents magnitude-squared bicoherence. Note that the topographic plots have different scale. The strongest bicoherence values appeared over occipito-parietal sensors. Grand mean frequency-by-frequency bicoherence comodulogram (middle) of occipital axial gradiometers of interest chosen from Figure S2A. Black rectangles indicate the x-y frequency selections used in the statistical analysis and topographic representations (8–12 Hz; 8–12 Hz; 18–22 Hz). Comodulograms have the same scale as alpha–alpha topography (left). Panels (B,C) are the same as (A) but for 0.5 and 1.5 mg LZP, respectively. [file Image6.TIF]

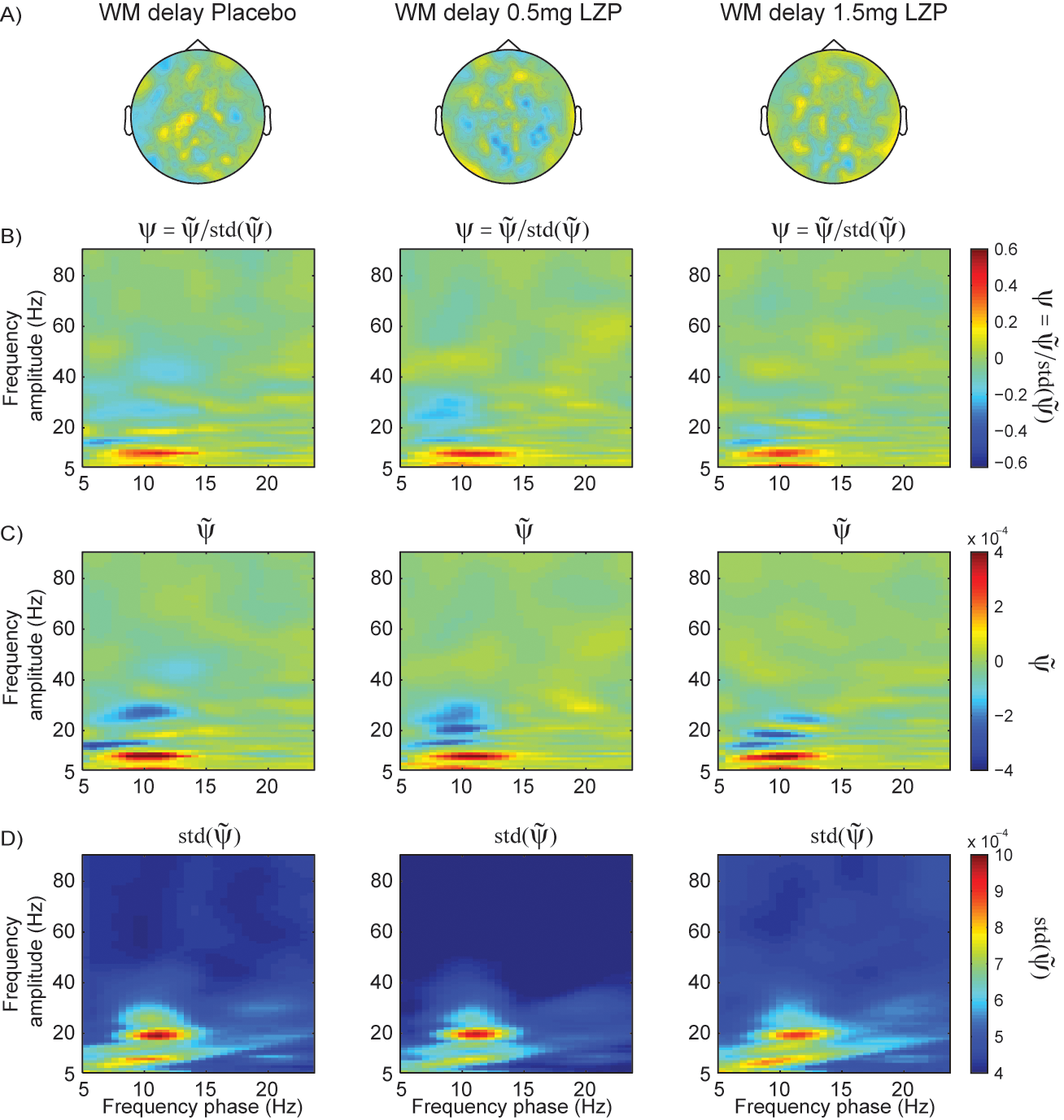

Supplement: Figure S7 — LZP did not change CFD during WM delay. (A) Grand mean topographic representation of alpha-beta CFD (16–22 Hz) during WM delay for each drug separately. Color code indicates the normalized Ψ~ weighted average of the CFD slope [Ψ = Ψ~/std(Ψ~); color scale same as in (B)]. (B) Grand mean normalized CFD index taken over the occipital sensors of interest chosen from Figure S2A. Note the low sigma values we obtained (~ ±0.6; ±2 is considered significant). (C) CFD weighted average (Ψ~) and (D) standard deviation of the slope (std(Ψ~)) taken over the same occipital sensors for each drug session respectively. Drug sessions are displayed in different columns. [file Image7.TIF]
